# Health facility service availability and readiness for intrapartum and immediate postpartum care in Malawi: A cross-sectional survey

**DOI:** 10.1371/journal.pone.0172492

**Published:** 2017-03-16

**Authors:** Naoko Kozuki, Lolade Oseni, Angella Mtimuni, Reena Sethi, Tambudzai Rashidi, Fannie Kachale, Barbara Rawlins, Shivam Gupta

**Affiliations:** 1 Department of International Health, Johns Hopkins Bloomberg School of Public Health Baltimore, Maryland, United States of America; 2 Jhpiego, Lilongwe, Malawi; 3 USAID, Lilongwe, Malawi; 4 Jhpiego, Washington, DC, United States of America; 5 Malawi Ministry of Health, Lilongwe, Malawi; Centre Hospitalier Universitaire Vaudois, FRANCE

## Abstract

This analysis seeks to identify strengths and gaps in the existing facility capacity for intrapartum and immediate postpartum fetal and neonatal care, using data collected as a part of Malawi’s Helping Babies Breath program evaluation. From August to September 2012, the Maternal and Child Health Integrated Program (MCHIP) conducted a cross-sectional survey in 84 Malawian health facilities to capture current health facility service availability and readiness and health worker capacity and practice pertaining to labor, delivery, and immediate postpartum care. The survey collected data on availability of equipment, supplies, and medications, and health worker knowledge and performance scores on intrapartum care simulation and actual management of real clients at a subset of facilities. We ran linear regression models to identify predictors of high simulation performance of routine delivery care and management of asphyxiated newborns across all facilities surveyed. Key supplies for infection prevention and thermal care of the newborn were found to be missing in many of the surveyed facilities. At the health center level, 75% had no clinician trained in basic emergency obstetric care or newborn care and 39% had no midwife trained in the same. We observed that there were no proportional increases in available transport and staff at a facility as catchment population increased. In simulations of management of newborns with breathing problems, health workers were able to complete a median of 10 out of 16 tasks for a full-term birth case scenario and 20 out of 30 tasks for a preterm birth case scenario. Health workers who had more years of experience appeared to perform worse. Our study provides a benchmark and highlights gaps for future evaluations and studies as Malawi continues to make strides in improving facility-based care. Further progress in reducing the burden of neonatal and fetal death in Malawi will be partly predicated on guaranteeing properly equipped and staffed facilities in addition to ensuring the presence of skilled health workers.

## Introduction

Malawi is one of two sub-Saharan African countries that successfully met its Millennium Development Goal #4 of reducing the national under-5 mortality rate by two-thirds between 1990 and 2015. The under-5 mortality rate in Malawi dropped from 227 in 1990 to 71 in 2013, with its target at 76 in 2015 [1]. In comparison, the neonatal mortality rate has experienced a slower rate of decline; neonatal health did not become a focal point until around 2005 [1]. Malawi has made various high-profile efforts to address neonatal death. The Community-Based Maternal and Newborn Care (CB-MNC) package utilizes the community health worker cadre of Health Surveillance Assistants to promote healthy pregnancy-related behaviors and to refer pregnant women and infants presenting danger signs. Kangaroo mother care (KMC) and Helping Babies Breathe (HBB) programs have been introduced in facilities across the country to address the burden of preterm birth and intrapartum-related hypoxia respectively [2]. While at a rate slower than the national reduction in under-5 mortality, neonatal mortality has been falling in Malawi at 3.5% per year between 2000 and 2010, over double the pace of the regional average (1.5% per year) [2]. The neonatal mortality rate was 22 per 1000 live births for 2015 estimates [2].

The reduction has been slower for stillbirths, with the Malawi national stillbirth rate dropping at the rate of 1.8% per year between 2000 and 2015, from a rate of 28.7 per 1000 births to 21.8 per 1000 births [3]. While half of all stillbirths are thought to occur in the intrapartum period globally, the proportion attributable to intrapartum causes is thought to be higher in low-resource settings such as sub-Saharan Africa [4]. At the global level, there are now more estimates available on the burden of long-term morbidities resulting from intrapartum complications in low-income settings [5]. For instance, with neonatal encephalopathy, which is largely thought to occur from intrapartum complications in a low-resource context, an estimated 287,000, 233,000, and 181,000 babies annually experience death, moderate-to-severe neurodevelopmental impairment, or mild impairment respectively [5]. Much of this is preventable by having skilled attendants with basic equipment at birth.

Coverage of facility delivery is high in Malawi (73%) [2], and with greater efforts through programs like CB-MNC, KMC, and the HBB program, there is great potential to further reduce not just neonatal mortality but also stillbirths and neonatal morbidities occurring in the intrapartum and immediate postpartum periods. Zimba et al. predicted in a 2012 publication that 16,000 neonatal deaths and 12,000 stillbirths could be averted in 2015 if neonatal interventions were to reach all families in Malawi, and that an additional 9000 lives could be saved with improvements in quality of facility-based care [2]. This paper seeks to examine facility service availability and readiness for, and health care worker knowledge of, intrapartum and immediate postpartum care in the country. A representative sample of 94 health centers and hospitals was surveyed as a part of the HBB national program evaluation. The evaluation collected data on equipment, supplies, medications, and transport available for maternal and neonatal care, as well as health worker availability and capacity. Based on those data, we identify strengths and gaps in the existing facility readiness and health worker capacity and provide recommendations for future efforts in reducing intrapartum-related mortality and morbidity in Malawi.

## Materials and methods

From August to September 2012, the Maternal and Child Health Integrated Program (MCHIP) conducted a cross-sectional survey of Malawian facilities to capture current health facility capacity pertaining to labor, delivery, and immediate postpartum care. The facility-based surveys presented here belonged to the first of two evaluation data collection stages following the rollout of the HBB program [6], an educational program that trains health workers on basic neonatal resuscitation techniques. The goal of the program is to have at least one individual trained in neonatal resuscitation available at the birth of every newborn. The survey consisted of facility inventory on equipment, supplies, and medications, as well as staff enumeration by health worker level and neonatal care training. The facility inventory questionnaire was adapted from Service Provision Assessment (SPA) surveys [7].

Separately, newborn care health workers at each facility were asked about their knowledge of equipment necessary for neonatal care, knowledge of tasks related to immediate newborn care, and knowledge of signs of neonatal sepsis. Names of newborn care health workers retained at each facility were documented as part of the facility assessment. In 20 of the larger facilities, a random sample of up to three health workers was interviewed. In the 70 remaining facilities, a random sample of up to two newborn care health workers was interviewed. A total of 220 health workers were interviewed. Of those 220, 191 health workers who had already received HBB training participated in directly observed simulations of intrapartum care, using an anatomic model, to test their capacity. They were given two case scenarios, one to handle a full-term delivery with breathing problems and one to handle a preterm delivery with breathing complications, using a newborn anatomic model. The health workers were expected to complete 16 tasks in the former and 30 tasks in the latter, and they were scored based on how many of those tasks they successfully completed. Three health workers were unable to partake in the second simulation, making the total 188 health workers. The list of the knowledge questions and answers as well as the list of simulation tasks the health workers were compared against is available in S1 and S2 Tables. The health worker knowledge questions and the simulation case scenario observation checklists were adapted from the SPAs, the World Health Organization’s “Managing Complications in Pregnancy and Childbirth” guide [8], and the Helping Babies Breathe facilitator tools [9]. Written informed consent was obtained from each participating individual.

A representative sample, totaling 90 facilities, was initially randomly selected from the 28 districts of Malawi. Four of the facilities were replaced when they yielded too few births for observation and six facilities were closed down between sampling and data collection, resulting in 84 facilities contributing data. A total of 24 hospitals and 60 health centers participated in the study. Of the 24 hospitals surveyed in this study, 20 were district hospitals, three were Christian Health Association of Malawi hospitals, and one was a private hospital. The catchment population for each facility varied widely. Among hospitals, the catchment population ranged from 5700 to 2.2 million, the latter being the central hospital in the capital, Lilongwe, with the median of roughly 50,000 people. Among the health centers, catchment area populations ranged from as few as 300 people to 588,000, with a median of 23,500 people (Table 1).

**Table 1:** Facilities surveyed in the study.

Facility type	Catchment population^a^	Expected number of women of reproductive age^a^
Hospital (n = 24)	• Median: 50000 • IQR: 25800–301900 • Range: 5700–2203911	• Median: 11000 • IQR: 5100–57500 • Range: 1700–506900
Health center (n = 56)	• Median: 23500 • IQR: 15100–37300 • Range: 300–588000	• Median: 4600 • IQR: 2000–6900 • Range: 0–44000

^a^rounded to nearest hundred.

Data on availability of equipment, supplies, and medications were collected as a binary variable of availability rather than the quantity of available items, and data on number of staff were collected and tabulated. We also summarized data on health workers’ knowledge and their performance scores on immediate postpartum care simulation. We ran linear regression models, using these performance scores as the dependent variable, and included as independent variables facility type, type of health worker, whether they had any training in labor and delivery, the number of years in delivery service, the number of years they had been involved in newborn care, whether they had received basic training in neonatal care in the last two years, the time at which they last received supervision, and their knowledge scores in necessary equipment for newborn care and in tasks for immediate postpartum care. For multivariate regression, we used the LASSO regression model that takes into account the collinearity between covariates and creates a more parsimonious model through variable selection.[10–12] The selected variables were refitted in a separate multi-level multiple linear regression model to account for regression coefficients that are biased toward zero in the Lasso regression. Stata version 12 was used for the analysis. The Institutional Review Boards of the Johns Hopkins Bloomberg School of Public Health and the University of Malawi’s College of Medicine Research and Ethics Committee approved the study.

## Results and discussions

Hospitals were generally well-stocked with necessary equipment and supplies in the labor ward for routine and complicated delivery care. A large majority (>90%) of the hospitals had a delivery bed for delivery, sterile gloves, a sharps container, already mixed decontaminating solution, and water for handwashing. However, there were key supplies for infection prevention like soap (67%) and hand disinfectant (18%) that were missing from a notable percentage of hospitals. Only 12.5% had one-time-use hand towels. Health centers had similar availability of equipment and supplies. Less than 50% of facilities had light for a pelvic exam (32%), hand disinfectant (20%), and single-use towels (10%), and only 75% of the health centers had soap visible in the labor ward (Table 2).

**Table 2:** Equipment and supplies in labor ward, in 24 hospitals and 60 health centers.

	Hospital			Health Center		
Equipment and supplies in labor ward	Observed, n (%)	Not observed, n (%)	TOTAL N	Observed, n (%)	Not observed, n (%)	TOTAL N
Spotlight for pelvic exam, flashlight/torch or exam light acceptable	16 (69.6)	7 (30.4)	23	19 (31.7)	41 (68.3)	60
Table or bed for delivery	24 (100.0)	0 (0.0)	24	69 (100.0)	0 (0.0)	60
Clean or sterile gloves	24 (100.0)	0 (0.0)	24	58 (96.7)	2 (3.3)	60
Sharps container	23 (95.8)	1 (4.2)	24	60 (100.0)	0 (0.0)	60
At least five or more 2-ml or 3-ml syringes (with 21-gauge needles)	21 (87.5)	3 (12.5)	24	57 (95.0)	3 (5.0)	60
Already mixed decontaminating solution	22 (91.7)	2 (8.3)	24	46 (76.7)	14 (23.3)	60
Hand disinfectant	4 (18.2)	18 (81.8)	22	12 (20.0)	48 (80.0)	60
Waste receptacle with lid and plastic liner	19 (79.2)	5 (20.8)	24	34 (57.6)	25 (42.4)	59
Soap for handwashing	16 (66.7)	8 (33.3)	24	45 (75.0)	15 (25.0)	60
Single-use hand-drying towel	3 (12.5)	21 (87.5)	24	6 (10.2)	53 (89.8)	59
Water for handwashing	22 (95.7)	1 (4.3)	23	59 (98.3)	1 (1.7)	60

Regarding the medications available, most facilities had supplies of intravenous solutions, parenteral antibiotics, uterotonics, and treatment for pre-eclampsia/eclampsia, although not all types of medication under each of these categories were available at all facilities. All hospitals and 90% of health centers had oxytocin available, the drug of choice for prevention of postpartum hemorrhage. Also, 100% and 92% of hospitals and 88% and 57% of health centers had injectable magnesium sulfate and anti-hypertensives available for management of pre-eclampsia/eclampsia respectively (Table 3).

**Table 3:** Medications in delivery area, in 24 hospitals and 60 health centers.

	Hospital			Health Center		
Medications in delivery area	Observed, n (%)	Not observed, n (%)	TOTAL N	Observed, n (%)	Not observed, n (%)	TOTAL N
Intravenous solutions: either Ringer’s lactate, D5NS, or NS infusion	24 (100.0)	0 (0.0)	24	60 (100.0)	0 (0.0)	60
Injectable ergometrine/methergine	2 (8.3)	22 (91.7)	24	2 (3.3)	58 (96.7)	60
Injectable oxytocin/syntocin	24 (100.0)	0 (0.0)	24	54 (90.0)	6 (10.0)	60
Misoprostol	10 (43.5)	13 (56.5)	23	2 (3.3)	58 (96.7)	60
Injectable diazepam	18 (75.0)	6 (25.0)	24	55 (91.7)	5 (8.3)	60
Injectable magnesium sulfate	24 (100.0)	0 (0.0)	24	53 (88.3)	7 (11.7)	60
Injectable amoxicillin or ampicillin	7 (31.8)	15 (68.2)	22	14 (23.7)	45 (76.3)	59
Injectable gentamicin	23 (95.8)	1 (4.2)	24	59 (98.3)	1 (1.7)	60
Calcium gluconate	2 (8.7)	21 (91.3)	23	3 (5.1)	56 (94.9)	59
Lidocaine (lignocaine)	24 (100.0)	0 (0.0)	24	58 (96.7)	2 (3.3)	60
Anti-hypertensives	22 (91.7)	2 (8.3)	24	33 (56.9)	25 (43.1)	58

For neonatal resuscitation, bag and mask (infant size, size 1) was available in nearly all facilities, but there was lower availability of the smaller infant size 0 mask for preterm babies (65% in hospitals, 68% in health centers) and tube and mask (78% in hospitals, 58% in health centers). The facilities were under-equipped to address thermoregulation and hypothermia prevention; only 54% of hospitals and 5% of health centers had incubators, 75% of hospitals and 12% of health centers had other sources of heat, and 42% of hospitals and 17% of health centers had a towel or blanket for wrapping the baby. Written guidelines for normal delivery and for HBB respectively were available in only about half of the hospitals and a quarter of the health facilities (Table 4).

**Table 4:** Equipment and supplies for newborn care, in 24 hospitals and 60 health centers.

	Hospital			Health center		
Equipment and supplies for newborn care	Observed, n (%)	Not observed, n (%)	TOTAL N	Observed, n (%)	Not observed, n (%)	TOTAL N
Bag and mask (infant size) for resuscitation–Size 0	15 (65.2)	8 (34.8)	23	41 (68.3)	19 (31.7)	60
Bag and mask (infant size) for resuscitation–Size 1	19 (100)	0 (0)	19	46 (95.8)	2 (4.2)	48
Tube and mask (infant size) for resuscitation	18 (78.3)	5 (21.7)	23	34 (57.6)	25 (42.4)	59
Resuscitation table for baby	22 (95.7)	1 (4.3)	23	40 (66.7)	20 (33.3)	60
Suction bulb for mucus extraction	16 (66.7)	8 (33.3)	24	35 (59.3)	24 (40.7)	59
Suction apparatus for use with catheter	20 (87.0)	3 (13.0)	23	48 (81.4)	11 (18.6)	59
Incubator	13 (54.2)	11 (45.8)	24	3 (5.0)	57 (95.0)	60
Other source of heat for premature infant	18 (75.0)	6 (25.0)	24	7 (11.9)	52 (88.1)	59
Towel or blanket to wrap baby	10 (41.7)	14 (58.3)	24	10 (16.9)	49 (83.1)	59
Infant scale	22 (91.7)	2 (8.3)	24	58 (96.7)	2 (3.3)	60
Disposable cord ties or clamps	23 (95.8)	1 (4.2)	24	50 (83.3)	10 (16.7)	60
Blank partographs	24 (100)	0 (0)	24	51 (86.4)	8 (13.6)	59
Guidelines for normal delivery	12 (54.5)	10 (45.5)	22	16 (27.1)	43 (72.9)	59
Guidelines for emergency obstetric care	23 (100)	0 (0)	23	48 (81.4)	11 (18.6)	59
Helping Babies Breathe guidelines for newborns not breathing at birth	14 (58.3)	10 (41.7)	24	15 (26.3)	42 (73.7)	57

In regard to transport, all hospitals owned at least one car, with the median number of vehicles at 5.5. Only 35% of health centers owned any car, and furthermore, only 58% of health centers owned at least one bicycle, motorcycle, or car (S3 Table). The median distance from health centers to the nearest referral facility was the same among health centers that owned at least one vehicle and those that did not; median distance was 35 km for those that did (IQR 24–53 km, range 5–100 km) and 30 km for those that did not (IQR 20–42 km, range 3–89 km).

Availability of trained clinical staff was low in the facilities, particularly those who had received any training in basic emergency obstetric care (BEmOC) or newborn care In hospitals, 12% did not have a single doctor (obstetrician or otherwise), medical assistant, or clinical officer allocated to the maternity, and 62% reported not having a single clinician trained in BEmOC or newborn care. All hospitals reported having at least three midwifery staff (which included registered midwife, enrolled nurse midwife, or midwife technician), but 15% of them reported not having a single midwife trained in BEmOC or newborn care. The dearth of personnel and lack of training were even more prominent in health centers; 13% had no clinician and 59% had one clinician allocated to the maternity ward, and 75% had no clinician and 23% had one clinician with BEmOC or neonatal care training. While all but one facility had at least one midwifery staff member in the maternity, 39% had no midwifery staff trained in BEmOC or neonatal care (S4 Table).

When data on availability of vehicles and staff (including those not trained in BEmOC or neonatal care) were stratified by the expected number of women of reproductive age in each facility’s catchment area, there was no obvious increase in vehicle and staff capacity with larger catchment populations. Even with a population of women of reproductive age in the 5000–10,000 range, there were still at least a quarter of the facilities that did not have any clinical or midwifery staff based in the maternity ward (S5 Table).

Each of the knowledge questions for the health workers had 11 possible answers, with a highest attainable score of 11. The health workers (n = 220) correctly reported a median of four (IQR 3–6, range 0–9) items of equipment needed for neonatal care, three (IQR 2–5, range 0–9) tasks that needed to be completed for immediate neonatal care, and four (IQR 3–4, range 0–8) signs of sepsis. Simulations were conducted to test health worker capacity in intrapartum and immediate postpartum care. In case scenario 1 (term neonate with difficulty breathing), the health workers had a median score of 10 completed tasks out of 16 (IQR 8–13, range 3–16).

The regression model for predictors of performance for a normal term delivery can be found in Table 5, the unit of the coefficients being tasks successfully completed. Health workers in a health center had slightly poorer performance than those in hospitals (-1.26, 95% CI: -2.41, -0.10), and those who received any basic training in neonatal health over the last two years (2.28, 95% CI: 1.08, 3.49) and those who were able to report more knowledge in necessary newborn care equipment (0.48, 95% CI: 0.17, 0.80) had slightly higher scores on performance.

**Table 5:** Regression model of health worker performance during intrapartum care simulation (normal, term delivery).

Variable		n (% or mean)	Coefficient (unit: tasks successfully completed) (95% CI)	p-value
Facility	Hospital	64 (40.3)	Ref	Ref
	Health center	95 (60.0)	-1.26 (-2.41, -0.10)	0.033
Years of service in delivery care	<1 yr	25 (14.7)	ref	Ref
	1–<2 yr	20 (11.8)	1.26 (-0.92, 3.44)	0256
	2–<5 yr	66 (38.8)	-0.01 (-2.38, 2.35)	0.991
	5–<20 yr	35 (20.6)	-1.89 (-5.24, 1.46)	0.267
	20+ yr	24 (14.1)	-2.01 (-6.53, 2.50)	0.379
Any basic training in delivery care in the last two years	No	87 (51.2)	Ref	Ref
	Yes	83 (48.8)	0.44 (-0.73, 1.62)	0.458
Any basic training in neonatal health in last 2 years	No	95 (59.8)	Ref	Ref
	Yes	64 (40.3)	2.28 (1.08, 3.49)	<0.001
Number of years in neonatal care	<1 yr	22 (13.8)	Ref	Ref
	1–<2 yr	42 (26.4)	0.66 (-1.24, 2.57)	0.49
	2–<5 yr	49 (30.8)	-0.85 (-2.80, 1.11)	0.40
	5–<20 yr	29 (18.2)	-1.77 (-5.14, 1.61)	0.30
	20+ yr	17 (10.7)	-2.13 (-7.25, 3.00)	0.41
When last supervised	< 3 months	78 (48.2)	1.53 (0.36, 2.71)	0.011
	3-<6 months	18 (11.1)	2.19 (0.31, 4.06)	0.022
	6+ months	21 (13.0)	1.04 (-0.67, 2.76)	0.231
	Not supervised	45 (27.8)	ref	Ref
Knowledge score pertaining to equipment needed for newborn care	Continuous	162 (4.4)	0.48 (0.17, 0.80)	0.003
Knowledge score pertaining to immediate newborn care	Continuous	162 (3.6)	0.08 (-0.30, 0.45)	0.680
Knowledge score pertaining to sepsis	Continuous	162 (3.8)	0.09 (-0.31, 0.48)	0.661

In case scenario 2 (preterm baby with breathing complications), which was conducted with 188 health workers, their median score was 20 completed tasks out of 30 tasks (IQR 13–23, range 3–30). Those who had received neonatal training in the last two years (2.62, 95% CI: 0.23 5.01), those who had recent supervision (reference: not supervised, <3 months, 3.43, 95% CI 1.01, 5.85, 3-<6 months, 5.68, 95% CI: 1.88, 9.49), registered or enrolled nurses / midwives (reference: clinical officers / medical assistants, 3.93, 95% CI 0.06, 7.81), and those who were able to report more knowledge in necessary newborn care equipment (1.05, 95% CI: 0.44, 1.66) had higher scores on performance. Health workers in health centers performed worse than those in hospitals (-2.81, 95% CI: -5.20, -0.41). Those who had over 20 years of experience performed worse by six tasks compared to those who had less than one year of experience (-7.38, 95% CI: -11,82, -2.94). (Table 6). The data files are available in S1 Data. No potentially participant-identifying information is within the files.

**Table 6:** Regression model of health worker performance during intrapartum care simulation (preterm delivery with complications).

Variable		n (% or mean)	Coefficient (unit: tasks successfully completed) (95% CI)	p-value
Facility	Hospital	93 (54.7)	Ref	Ref
	Health center	77 (45.3)	-2.81 (-5.20, -0.41)	0.022
Worker type	Clinical officer / medical assistant	24 (14.1)	Ref	Ref
	Registered / enrolled nurse / midwife	43 (25.3)	3.93 (0.06, 7.81)	0.047
	Nurse / midwife technician	103 (60.6)	3.21 (-0.07, 6.49)	0.055
Years of service in delivery care	<1 yr	24 (14.3)	Ref	Ref
	1–<2 yr	20 (11.9)	1.34 (-7.29, 9.98)	0.759
	2–<5 yr	66 (39.3)	-0.92 (-8.72, 6.89)	0.817
	5–<20 yr	34 (20.2)	-3.05 (-10.14, 4.04)	0.397
	20+ yr	24 (14.3)	-7.38 (-11.82, -2.94)	0.001
Any basic training in delivery care in the last two years	No	86 (51.2)	Ref	Ref
	Yes	82 (48.8)	0.17 (-2.19, 2.53)	0.888
Any basic training in neonatal care in last 2 years	No	64 (39.0)	Ref	Ref
	Yes	100 (61.0)	2.62 (0.23, 5.01)	0.032
Number of years in neonatal care	<1 yr	24 (14.3)	Ref	Ref
	1–<2 yr	45 (26.8)	-0.52 (-8.11, 7.08)	0.893
	2–<5 yr	53 (31.6)	-0.11 (-7.11, 6.90)	0.976
	5–<20 yr	28 (16.7)	1.63 (-4.54, 7.79)	0.603
	20+ yr	18 (10.7)	N/A	N/A
When last supervised	< 3 months	43 (26.9)	3.43 (1.01, 5.85)	0.006
	3-<6 months	79 (49.4)	5.68 (1.88, 9.49)	0.004
	6+ months	18 (11.3)	0.96 (-2.53, 4.46)	0.587
	Not supervised	20 (12.5)	Ref	Ref
Knowledge score pertaining to equipment needed for newborn care	Continuous	181 (4.0)	1.05 (0.44, 1.66)	0.001
Knowledge score pertaining to immediate newborn care	Continuous	181 (3.3)	0.43 (-0.28, 1.13)	0.236

Our study summarizes the resource availability and staff competencies related to facility-based intrapartum and immediate postpartum care in Malawi. Newborn asphyxia, which is affected by the quality of intrapartum care, is one of the leading causes of fetal and neonatal death in Malawi [1, 4]. Malawi’s Every Newborn Action Plan has highlighted as one of its four strategies the need to “strengthen and invest in care during labour, delivery, first day and week of life” to meet the goal of reducing neonatal mortality to 15 per 1000 live births by 2035 [13]. Further progress in reducing the burden of fetal and neonatal death in Malawi will be partly predicated on guaranteeing properly equipped and staffed facilities in addition to skilled health workers. The data presented here serve as a benchmark for future evaluations and studies as Malawi continues to make strides in improving facility-based care.

The Donabedian quality-of-care framework highlights structure, or the attributes of the care provision setting, as a key component of high quality health care. The facilities we examined here appeared to meet the structural demands in relation to equipment better than in relation to personnel and transport needs. Facilities generally had equipment and supplies to provide appropriate care for intrapartum and immediate postnatal complications. Medications were widely available for major clinical indications like pre-eclampsia/eclampsia, postpartum hemorrhage, and sepsis. Notable gaps in supplies included items for infection prevention and thermal control, which are both directly linked to sepsis and preterm birth, two of the major causes of neonatal death. Less than half of the facilities had towels or blankets available to wrap the newborn immediately after birth. These issues require improved supply chain management; there are ongoing supply chain management and integration programs that are using methods such as training Pharmacy Assistants, increasing commodity storage space using pre-fabricated storage units, and training Health Surveillance Assistants to serve as drug store clerks.[14] Building reporting capacity of MoH facilities in logistics management information systems will also aid Pharmacy Assistants make proper forecasts and acquire commodities for facilities in their districts. From our data, we were unable to assess the capacity of providers to initiate KMC among the facilities lacking thermal care equipment. While there has been a national emphasis on KMC, the practice has not been well-integrated in many facilities throughout the country. A 2012 evaluation of KMC services [15], in which 14 facilities were assessed, found that only five of the facilities had been deemed as having routine practice of KMC and none were deemed to have sustainable practice. Great efforts to improve KMC capacity have been made in the past several years through the ongoing implementation of the Support for Service Delivery Integration–Services (SSDI-Services) Project. It was reported that between Year 1 and Year 4 of SSDI-Services implementation, the number of facilities providing KMC services increased from 63 to 170, and all 15 participating districts receiving KMC supportive supervision [16]. Particularly in a country with the highest rate of preterm birth globally, improving thermal care capacity will be invaluable in reducing mortality and morbidities associated with babies born too soon. It is estimated that globally, 1.1 million neonatal deaths [17], 345,000 moderate-to-severe neurodevelopmental impairment cases, and 567,000 mild neurodevelopmental impairment cases [18] are attributable to preterm births annually.

Facilities were generally poorly staffed, with a substantial percentage of facilities having no midwife-level staff trained in BEmOC or neonatal care (15% of hospitals and 40% of health centers). Notably, the availability of staff in facilities did not increase proportionally, if at all, with the increase in catchment population. For example, the median number of midwifery-level staff trained in BEmOC or neonatal care remained at one as the number of women of reproductive age in respective catchment areas increased from 0–<2000 to 2000–<5000 to 5000–<10,000, with the median increasing only to two with the number increasing to 10,000–<50,000. A study conducted at a district hospital in Malawi showed great burnout rates among maternal health staff, with nearly three-quarters of the staff reporting emotional exhaustion, and concluded that burnout rates were higher among maternal health staff compared to other health workers [19]. Although the study did not explore workload or the worker-to-catchment-population ratio as predictors of burnout, the failure to staff the facilities based on expected workload could contribute to undue stress on facility staff and result in poor care. Malawi’s Every Newborn Action Plan has also identified as major bottlenecks the low number of health workers, poor staff retention, and inequitable staff deployment [13], which were also reflected in our quantitative data. This burden may only worsen, as an evaluation of CB-MNC has seen promising improvements in household knowledge of maternal and newborn danger signs and increases in institutional deliveries [20]. The Ministry of Health (MoH) can use data like what we present here to deploy health care workers where there are disproportionate gaps in personnel. Strengthening and updating the human resource information system in the MoH will also help to keep the MoH aware of staff movements across districts and facilities, and thereby inform appropriate deployment.

A significant proportion of health centers had no ownership of a vehicle, and we presume the lack of reliable access to transport would severely inhibit the ability of lower-level facilities to promptly refer women to referral facilities. Health centers with no ownership of a vehicle had a median distance of 35 km to the nearest referral facility, and the distance is expected to be longer if referral is being made to a comprehensive emergency obstetric care facility. The most recent Demographic and Health Survey conducted in 2010 reported a national cesarean section rate of 4.6% [21]; the reference provided by the United Nations is for a minimum of 5% and a maximum of 15% of deliveries to be conducted via cesarean section, based on the expected incidence of intrapartum complications [22]. The ability for health workers to properly refer during emergencies, and then the subsequent access to transport, are both critical for improving intrapartum outcomes.

In assessing the process indicators, relevant knowledge and receiving neonatal care training in the last two years were associated with better simulation performance. Our regression analysis suggested that the longer health workers had been working in neonatal care, the worse they performed, possibly suggesting that newer standards of care may need to be re-enforced with more experienced members of the staff. This may suggest that routine re-training programs may be able to improve staff performance, and old techniques and care provisions may need to be changed with the introduction of new guidelines. A process documentation of the HBB rollout conducted in 2013 also reported funding constraints contributing to equipment and training deficits [23]; our data, collected early in the HBB rollout period, may also be reflecting such limitations of the implementation. To better identify specific gaps in performance in the future, use of a standardized set of process indicators for quality of neonatal and maternal care may be beneficial. A study by Brenner et al. also conducted in Malawi stratified routine maternal and neonatal care process indicators into risk assessment, risk monitoring, and risk prevention, and observed that health care providers had the lowest adherence to clinical standards for risk monitoring. This allowed for more targeted intervention to improve the quality of care [24].

It is important to note that high performance as defined by knowledge and correct simulation does not necessarily correspond to good practice. For instance, in a separate analysis that compared the simulation data and direct observations conducted at the same facilities in the second round data collection for this evaluation, simulations corresponded with high performance for certain tasks, such as drying of the baby, but not for other tasks, such as cord clamping, placement of the newborn for skin-to-skin contact, and handwashing [25]. In a Tanzanian study that evaluated the effect of a one-day HBB training course, the investigators observed an increase in the number of providers who passed the simulation, yet no change was observed in actual clinical practice [26]. Additional data from facility observations are necessary to better evaluate the standard of intrapartum and immediate postpartum care.

## Conclusion

While Malawian health facilities generally had proper equipment and supplies to provide appropriate intrapartum and immediate postpartum care, there was a scarcity of health workers relative to the catchment population of a facility. There was also a trend of those reporting fewer years in neonatal care performing better in simulations than those with more years of neonatal care, hinting at the need for refresher training and also for involving established clinicians in discussions of new guidelines. The burden of fetal and neonatal mortality and morbidity attributable to intrapartum-related complications is still high in Malawi. These gaps in quality of facility-based care will need to be revisited to make further progress in reducing the burden.

## Supporting information

S1 Table Questions used for health worker knowledge assessment of neonatal care. (DOCX)

S2 Table Tasks on which health workers were assessed in intrapartum simulations. (DOCX)

S3 Table Access to transportation, 24 hospitals and 60 health centers. (DOCX)

S4 Table Number of health workers at surveyed facilities. (DOCX)

S5 Table Number of vehicles and staff, by catchment population of facilities. (DOCX)

S1 Data Data used in the analyses. (ZIP)

## References

[1] Countdown to 2015. Countdown Country Case Study: Malawi. 2015.

[2] Zimba E, Kinney MV, Kachale F, Waltensperger KZ, Blencowe H, Colbourn T, et al Newborn survival in Malawi: a decade of change and future implications. Health Policy Plan. 2012;27 Suppl 3:iii88–103. Epub 2012/08/09. 22692419 10.1093/heapol/czs043

[3] Blencowe H, Cousens S, Jassir F, Say L, Chou D, Mathers C, et al National, regional, and worldwide estimates of stillbirth rates in 2015, with trends from 2000: a systematic analysis. Lancet Global Health. 2016. Epub 18 January 2016. 10.1016/S2214-109X(15)00275-2 26795602

[4] Lawn JE, Blencowe H, Pattinson R, Cousens S, Kumar R, Ibiebele I, et al Stillbirths: Where? When? Why? How to make the data count? Lancet. 2011;377(9775):1448–63. Epub 2011/04/19. 10.1016/S0140-6736(10)62187-3 21496911

[5] Lee AC, Kozuki N, Blencowe H, Vos T, Bahalim A, Darmstadt GL, et al Intrapartum-related neonatal encephalopathy incidence and impairment at regional and global levels for 2010 with trends from 1990. Pediatr Res. 2013;74 Suppl 1:50–72. Epub 2013/12/25. PubMed Central PMCID: PMC3873711. 24366463 10.1038/pr.2013.206 PMC3873711

[6] American Academy of Pediatrics. Helping Babies Breathe [cited 2014 Feb 2]. Available from: http://www.helpingbabiesbreathe.org/.

[7] Demographic and Health Survey. SPA Overview. Available from: http://dhsprogram.com/What-We-Do/Survey-Types/SPA.cfm.

[8] World Health Organization. Managing complications in pregnancy and childbirth: a guide for midwives and doctors. Geneva: 2007.

[9] American Academy of Pediatrics. Helping Babies Breathe: Facilitator Tools Elk Grove Village, Illinois: American Academy of Pediatrics; [cited 2016 20 January]. Available from: http://www.helpingbabiesbreathe.org/facilitatortools.html.

[10] Tibshirani R. Regression Shrinkage and Selection via the Lasso. Journal of the Royal Statisical Society. 1996;58(1):267–88.

[11] Goggin K, Wexler C, Nazir N, Staggs VS, Gautney B, Okoth V, et al Predictors of Infant Age at Enrollment in Early Infant Diagnosis Services in Kenya. AIDS Behav. 2016;20(9):2141–50. PubMed Central PMCID: PMCPMC4995224. 10.1007/s10461-016-1404-z 27108002 PMC4995224

[12] van Lettow M, Bedell R, Landes M, Gawa L, Gatto S, Mayuni I, et al Uptake and outcomes of a prevention-of mother-to-child transmission (PMTCT) program in Zomba district, Malawi. BMC Public Health. 2011;11:426 PubMed Central PMCID: PMCPMC3126744. 10.1186/1471-2458-11-426 21639873 PMC3126744

[13] Malawi Ministry of Health. Every Newborn Action Plan: an action plan to end preventable neonatal deaths in Malawi.

[14] USAID. USAID Malawi Health Commodity Supply Chain Fact Sheet 2015. Available from: https://www.usaid.gov/malawi/fact-sheets/usaid-malawi-supply-chain-fact-sheet-2012-13.

[15] Berg A BL, Lipato T, Ngwira G, Luhanga R, Ligowe R. Evaluation of Kangaroo Mother Care services in Malawi. 2012.

[16] Wendo D, Bartlett P, Oseni L, Kalemba N. Support for Service Delivery Integration FY15 Annual Report (Malawi). 2015.

[17] Liu L JH, Cousens S, Perin J, Scott S, Lawn JE, Rudan I, Campbell H, Cibulskis R, Li M, Mathers C, Black RE; Child Health Epidemiology Reference Group of WHO and UNICEF. Global, regional, and national causes of child mortality: an updated systematic analysis for 2010 with time trends since 2000. Lancet. 2012;379:2151–61. 10.1016/S0140-6736(12)60560-1 22579125

[18] Blencowe H, Lee AC, Cousens S, Bahalim A, Narwal R, Zhong N, et al Preterm birth-associated neurodevelopmental impairment estimates at regional and global levels for 2010. Pediatr Res. 2013;74 Suppl 1:17–34. Epub 2013/12/25. PubMed Central PMCID: PMC3873710. 24366461 10.1038/pr.2013.204 PMC3873710

[19] Thorsen VC, Tharp AL, Meguid T. High rates of burnout among maternal health staff at a referral hospital in Malawi: A cross-sectional study. BMC Nurs. 2011;10:9 Epub 2011/05/25. PubMed Central PMCID: PMC3114002. 10.1186/1472-6955-10-9 21605379 PMC3114002

[20] Callaghan-Koru JA, Nonyane BA, Guenther T, Sitrin D, Ligowe R, Chimbalanga E, et al Contribution of community-based newborn health promotion to reducing inequities in healthy newborn care practices and knowledge: evidence of improvement from a three-district pilot program in Malawi. BMC public health. 2013;13:1052 PubMed Central PMCID: PMCPMC3833651. 10.1186/1471-2458-13-1052 24199832 PMC3833651

[21] National Statistical Office (Malawi), ICF Macro. Malawi Ddemographic and Health SUrvey, 2010. Zomba, Malawi, and Calverton, Maryland: 2010.

[22] Appropriate technology for birth. Lancet. 1985;2(8452):436–7. Epub 1985/08/24. 2863457

[23] McPherson RA. A Process Documentation of the Scale-Up of the Helping Babies Breathe Initiative in Malawi 2013.

[24] Brenner S, De Allegri M, Gabrysch S, Chinkhumba J, Sarker M, Muula AS. The quality of clinical maternal and neonatal healthcare—a strategy for identifying 'routine care signal functions'. PLoS One. 2015;10(4):e0123968 PubMed Central PMCID: PMCPMC4398438. 10.1371/journal.pone.0123968 25875252 PMC4398438

[25] Sethi R. The measurement of the quality of maternal and newborn care in Malawi: comparative analyses of health worker performance measurement methods and an exploration of evidence of respectful maternity care. Baltimore: Johns Hopkins University; 2015.

[26] Ersdal HL, Vossius C, Bayo E, Mduma E, Perlman J, Lippert A, et al A one-day "Helping Babies Breathe" course improves simulated performance but not clinical management of neonates. Resuscitation. 2013;84(10):1422–7. 10.1016/j.resuscitation.2013.04.005 23612024

