# Supplementary material for: Health facility service availability and readiness for intrapartum and immediate postpartum care in Malawi: A cross-sectional survey
Source: PLoS One. 2017 Mar 16;12(3):e0172492. doi: 10.1371/journal.pone.0172492 (PMC5354363; doi:10.1371/journal.pone.0172492)
Supplement: S1 Table — (DOCX) [file pone.0172492.s001.docx]

**S1 Table.** Questions used for health worker knowledge assessment of neonatal care

| Question | Acceptable answers (one point given for each of the following) |
| --- | --- |
| What basic equipment and supplies must be available to ensure the baby receives appropriate immediate care after birth? | 2 dry warm towels or cloths, sterile blade or scissors, sterile or disposable cord ties/ clamps, cap for baby, source of warmth (heating lamp or incubator), self-inflating ventilation bag, newborn face mask size 1, newborn face mask size 0, mucus extractor/suction/bulb syringe, flat surface, clock or watch with seconds  (11 total) |
| When a baby is delivered and there is no complication, what care is important to give the baby immediately after birth and in the first hour? | Wipe face after birth of head, ensure baby was breathing/crying, provide thermal protection – place skin to skin with mother, provide thermal protection – wrap baby in a towel/cloth, suction newborn with bulb, ensure mother initiates breastfeeding within 1 hour, assess/examine newborn within 1 hour, weigh newborn, provide eye prophylaxis/antibiotic ointment, cut cord with sterile blade/scissors, apply antiseptic or other material to cord stump  (11 total) |
| Can you please tell me the signs and symptoms of severe infection (sepsis) in a newborn? | Poor/no breastfeeding, restlessness/irritability, breathing difficulties, hypothermia, hyperthermia, breathing rate >60/minute, convulsions, pus/redness around umbilicus, abscess on any part of body, skin pustules, lethargy/no movement (but conscious), unconscious  (12 total) |
