# Supplementary material for: Health facility service availability and readiness for intrapartum and immediate postpartum care in Malawi: A cross-sectional survey
Source: PLoS One. 2017 Mar 16;12(3):e0172492. doi: 10.1371/journal.pone.0172492 (PMC5354363; doi:10.1371/journal.pone.0172492)
Supplement: S2 Table — (DOCX) [file pone.0172492.s002.docx]

**S2 Table.** Tasks on which health workers were assessed in intrapartum simulations

| **Case scenario 1** | **Case scenario 2** |
| --- | --- |
| ***Prepares for birth*** | ***Prepares for birth*** |
| Identifies a helper | Identifies a helper |
| Makes an emergency plan | Prepares area for delivery |
| Prepares area for delivery | Cleans hands |
| Cleans hands and maintains clean technique throughout | Prepares an area for ventilation |
| Prepares an area for ventilation | Checks equipment |
| Checks equipment | ***Keeps baby warm*** |
| ***Keeps baby warm*** | Dries thoroughly |
| Dries thoroughly | Removes wet cloth |
| Removes wet cloth | Covers baby with dry cloth |
| Covers baby with dry cloth | ***Evaluates crying*** |
| ***Evaluates crying*** | Recognizes baby is not crying |
| Recognizes baby is not crying | ***Clears airway and stimulates breathing*** |
| ***Clears airway and stimulates breathing*** | Positions head and clears airway |
| Positions head and clears airway | Clears airway |
| Simulates breathing by rubbing the back | Simulates breathing by rubbing the back |
| ***Evaluates breathing*** | ***Evaluates breathing*** |
| Recognizes baby is breathing well | Recognizes baby is not breathing |
| Clamps or ties and cuts cord | ***Ventilates with bag and mask*** |
| Positions skin-to-skin on mother's chest | Cuts cord |
| Communicates with mother | Moves to area for ventilation |
|  | Starts ventilation within the Golden Minute |
|  | Ventilates at 40 breaths per minute |
|  | Looks for chest movement |
|  | ***Evaluates breathing*** |
|  | Recognizes baby is not breathing |
|  | Calls for help |
|  | Continues ventilation |
|  | ***Improves ventilation*** |
|  | Head – repositions neck |
|  | Reapplies mask |
|  | Mouth – clears secretion, opens mouth slightly |
|  | Bag – squeezes bag harder |
|  | ***Evaluates breathing and heart rate*** |
|  | Recognizes baby is breathing |
|  | Stops ventilation |
|  | Monitors baby |
|  | Communicates with mother |
