# Supplementary material for: Health facility service availability and readiness for intrapartum and immediate postpartum care in Malawi: A cross-sectional survey
Source: PLoS One. 2017 Mar 16;12(3):e0172492. doi: 10.1371/journal.pone.0172492 (PMC5354363; doi:10.1371/journal.pone.0172492)
Supplement: S3 Table — (DOCX) [file pone.0172492.s003.docx]

**S3 Table.** Access to transportation, 24 hospitals and 60 health centers

| Access to transportation | | Hospital | | Health center | |
| --- | --- | --- | --- | --- | --- |
|  |  | Y | N | Y | N |
| Bicycle | Number of facilities | 1 (7 %) | 13 (93 %) | 4 (11 %) | 32 (89 %) |
|  | Number of bicycles | median 0, range 0–2 | | median 0, range 0–37 | |
| Motorcycle | Number of facilities | 11 (73 %) | 4 (27 %) | 21 (51 %) | 20 (49 %) |
|  | Number of motorcycles | median 10, range 0–53 | | median 1, range 0–6 | |
| Car | Number of facilities | 18 (100 %) | 0 (0 %) | 15 (35 %) | 28 (65 %) |
|  | Number of cars | median 5.5, range 1–17 | | median 0, range 0–8 | |
| At least one of the above | Number of facilities | 18 (100 %) | 0 (0 %) | 32 (58 %) | 23 (42 %) |
