# Supplementary material for: Health facility service availability and readiness for intrapartum and immediate postpartum care in Malawi: A cross-sectional survey
Source: PLoS One. 2017 Mar 16;12(3):e0172492. doi: 10.1371/journal.pone.0172492 (PMC5354363; doi:10.1371/journal.pone.0172492)
Supplement: S4 Table — (DOCX) [file pone.0172492.s004.docx]

**S4 Table.** Number of health workers at surveyed facilities

| Cadre of maternal and newborn health service providers at health facility | # of providers at the facility | Hospital | | | | Health center | | | |
| --- | --- | --- | --- | --- | --- | --- | --- | --- | --- |
|  |  | Number allocated to maternity | | Number trained in BEmOC or newborn care | | Number allocated to maternity | | Number trained in BEmOC or newborn care | |
|  |  | # of facilities | % | # of facilities | % | # of facilities | % | # of facilities | % |
| Any doctor, medical assistant, or clinical officer | 0 | 3 | 11.5 | 13 | 61.9 | 7 | 13.2 | 36 | 75 |
|  | 1 | 4 | 15.4 | 5 | 23.8 | 31 | 58.5 | 11 | 22.9 |
|  | 2 | 2 | 7.7 | 0 | 0 | 9 | 16.9 | 1 | 2.1 |
|  | 3 | 7 | 26.9 | 1 | 4.8 | 1 | 1.9 | 0 | 0 |
|  | 4 | 8 | 30.8 | 2 | 9.5 | 3 | 5.7 | 0 | 0 |
|  | 5 | 2 | 7.7 | 0 | 0 | 2 | 3.8 | 0 | 0 |
| Registered midwife, enrolled nurse midwife, or midwife technician | 0 | 0 | 0.0 | 4 | 14.8 | 1 | 1.9 | 19 | 38.8 |
|  | 1 to 2 | 0 | 0.0 | 8 | 29.6 | 34 | 65.4 | 26 | 53.1 |
|  | 3 to 6 | 6 | 20.0 | 3 | 11.1 | 7 | 13.5 | 4 | 9.2 |
|  | 7 to 10 | 8 | 26.7 | 9 | 33.4 | 4 | 7.7 | 0 | 0 |
|  | 11+ | 16 | 53.3 | 3 | 11.1 | 6 | 11.5 | 0 | 0 |
