# Supplementary material for: Health facility service availability and readiness for intrapartum and immediate postpartum care in Malawi: A cross-sectional survey
Source: PLoS One. 2017 Mar 16;12(3):e0172492. doi: 10.1371/journal.pone.0172492 (PMC5354363; doi:10.1371/journal.pone.0172492)
Supplement: S5 Table — (DOCX) [file pone.0172492.s005.docx]

**\**

**S5 Table.** Number of vehicles and staff, by catchment population of facilities

| Expected # of women of reproductive age in respective catchment areas (n = # of facilities) |  | Motorcycle (n) | Car (n) | Any medical staff (n) | Any midwifery staff (n) | Medical staff trained in newborn care (n) | Midwifery staff trained in newborn care (n) |
| --- | --- | --- | --- | --- | --- | --- | --- |
| 0–<2000 (n=16) | Median | 1 | 0 | 1 | 1.5 | 0 | 1 |
|  | IQR | 0–1 | 0–0.5 | 1–1 | 1–3.5 | 0–0 | 0–1 |
|  | Range | 0–4 | 0–5 | 0–4 | 1–11 | 0–1 | 0–2 |
| 2000–<5000 (n=18) | Median | 0.5 | 0 | 1 | 2 | 0 | 1 |
|  | IQR | 0–5.5 | 0–6 | 1–2 | 2–3 | 0–1 | 0–2 |
|  | Range | 0–38 | 0–12 | 0–4 | 0–12 | 0–1 | 0–7 |
| 5000–<10000 (n=23) | Median | 1 | 1 | 1 | 3 | 0 | 1 |
|  | IQR | 0–1 | 0–1 | 1–2.5 | 2–10 | 0–0 | 0–2 |
|  | Range | 0–40 | 0–13 | 0–5 | 1–14 | 0–1 | 0–9 |
| 10000–<50000 (n=14) | Median | 0 | 1 | 2.5 | 9.5 | 0 | 2 |
|  | IQR | 0–2 | 0–1 | 1–4 | 5–14 | 0–1 | 1–6 |
|  | Range | 0–53 | 0–17 | 0–5 | 2–23 | 0–4 | 0–10 |
| 50000–<100000 (n=5) | Median | 9 | 7 | 4.5 | 10 | 2 | 7 |
|  | IQR | 2–16 | 4.5–9.5 | 4-5 | 9–11 | 0–4 | 5–8 |
|  | Range | 2–16 | 3–11 | 4-5 | 7–12 | 0–4 | 1–11 |
| 100000+ (n=4) | Median | 0 | 4 | 10 | 18 | 1 | 10.5 |
|  | IQR | 0 | 1–7 | 9–11 | 13–23.5 | 1–3 | 9–11 |
|  | Range | 0 | 1-7 | 7–12 | 11–26 | 1–3 | 8–11 |
