# Supplementary material for: Health facility service availability and readiness for intrapartum and immediate postpartum care in Malawi: A cross-sectional survey
Source: PLoS One. 2017 Mar 16;12(3):e0172492. doi: 10.1371/journal.pone.0172492 (PMC5354363; doi:10.1371/journal.pone.0172492)
Supplement: S1 Data — (ZIP) [file pone.0172492.s006.zip › Malawi HBB Eval Tool 1_HWI and knowledge test August 18 FINAL.docx]

**MALAWI HBB EVALUATION**

**Tool 1: Health Worker Interview & Knowledge Test**

| Cover Sheet | | | |
| --- | --- | --- | --- |
| H1: Facility name |  | **H2: Facility number** | **\|__C/I__\|____\|____\|____\|** |
| H3: Observer number | **\|____\|____\|** | **H4: Today’s date**  **DD/MM/YY** | **\|__D__\|__D__\|__M__\|__M__\|__Y__\|__Y__\|** |
| *Explain to the health worker that his/her name was provided as a knowledgeable maternal and/or neonatal health provider available on that day. Validate with the health worker that he/she does provide some maternal and/or neonatal health services in this facility. If this is a new study participant, obtain informed consent. If the person is not a new respondent, Proceed to H6.* | | | |
| H6: Health worker line number (from staff listing) | **\|___\|___\|** | **H7: Sex of health worker** | **Male 1** |
|  |  |  | **Female 2** |

| Section 1: Interview | | | | |
| --- | --- | --- | --- | --- |
| ***Read the following questions to the health worker. If health worker doesn’t know the year, probe using past events and record your best estimate.*** | | | | |
| **Question** | **Code** |  | |  |
| **Section 1: EDUCATION AND EXPERIENCE** |  |  | |  |
| **H8: What is your current professional/technical/medical qualification?** |  |  | |  |
| **Obstetrician/Gynaecologist** | **1** |  | |  |
| **Doctor** | **2** |  | |  |
| **Medical Assistant** | **3** |  | |  |
| **Clinical Officer** | **4** |  | |  |
| **Registered Midwife** | **5** |  | |  |
| **Enrolled Nurse/Midwife** | **6** |  | |  |
| **Nurse/Midwife Technician** | **7** |  | |  |
| **Student** | **8** |  | |  |
| **Other** | **9** |  | |  |
|  |  |  | |  |
| **H100: What year did you graduate (or complete) with this qualification?** | **\|___\|___\|___\|___\|** | | | |
| **H101: In what year did you start working in this facility?** | **\|___\|___\|___\|___\|** | | | |
| **H102: In what year did you start working in your current position in this facility?** | **\|___\|___\|___\|___\|** | | | |
| **H103: What is your age? *(Observer: if health worker doesn’t want to give age, ask them to give you a range, i.e. between age 40 and 50. If they refuse to give an age range, enter “00” in the “Age in years” boxes)*** | **Age in years:**  **Age range:**  **< 18 years of age**  **18-24**  **25-34**  **35-44**  **45-54**  **55-64**  **65+** | | **\|______\|_______\|**  **1**  **2**  **3**  **4**  **5**  **6**  **7** | |
| **TRAINING AND SERVICES PROVIDED** |  |  | |  |
| **Question** | **Yes** | **No** | | **Go to** |
|  |  |  | |  |
| **H106: During the past 2 years have you received any pre-service (basic) or in-service training on subjects related to antenatal care?** | **1** | **0** | | **No→H108** |
| **H107: In the past 2 years, did you receive any training on the following topics *()*:** | **1** | **0** | |  |
| 1. **ANC screening (e.g., blood pressure, urine glucose and protein)?** | **1** | **0** | |  |
| 1. **Counseling for ANC (e.g., nutrition, FP and newborn care)?** | **1** | **0** | |  |
| 1. **Emergency obstetric and newborn care (EmONC)** | **1** | **0** | |  |
| 1. **Management of pre-eclampsia/eclampsia** | **1** | **0** | |  |
| 1. **Any topic related to pregnancy and HIV/AIDS or PMTCT?** | **1** | **0** | |  |
| **H108: In your current position, and as a part of your work for this facility, do you personally provide any delivery services? By that I mean conducting the actual delivery of newborns** | **1** | **0** | | **No→H114** |
| **H109: How many years in total have you provided such services? Service may have been here or in another facility *(Observer: enter 00 if less than 1 year of service)*** | **\|___\|___\|** | | |  |
| **Question** | **Code** |  | |  |
| **H110: How often do you use a partograph*(read each answer aloud)*:** |  |  | |  |
| **Never** | **1** |  | |  |
| **Rarely** | **2** |  | |  |
| **Sometimes** | **3** |  | |  |
| **Most of the time** | **4** |  | |  |
| **Always** | **5** |  | |  |
| **H111: How often do you use active management of the third stage of labor (AMTSL) during normal vaginal births *(read each answer aloud)*:** |  |  | |  |
| **Never** | **1** |  | |  |
| **Rarely** | **2** |  | |  |
| **Sometimes** | **3** |  | |  |
| **Most of the time** | **4** |  | |  |
| **Always** | **5** |  | |  |
| **Question** | **Yes** | **No** | | **Go to** |
| **H112: During the past 2 years have you received any pre-service (basic) or in-service training on subjects related to delivery care?** | **1** | **0** | | **No→H114** |
|  |  |  | |  |
| **H113: In the past 2 years, did you receive any training on the following topics *(read each answer aloud)*:** |  |  | |  |
| 1. **Routine care for labor and normal vaginal delivery** | **1** | **0** | |  |
| 1. **Use of partograph** | **1** | **0** | |  |
| 1. **Active management of third stage of labor (AMTSL)** | **1** | **0** | |  |
| 1. **Emergency obstetric care (EmOC)/Life saving skills (LSS) - in general** | **1** | **0** | |  |
| 1. **Management of sepsis, including use of parenteral antibiotics** | **1** | **0** | |  |
| 1. **Administer magnesium sulfate for the treatment of severe pre-eclampsia or eclampsia** | **1** | **0** | |  |
| 1. **Management of postpartum hemorrhage** | **1** | **0** | |  |
| 1. **Removal of placenta or products of conception? (D&C, vacuum aspiration, etc.)** | **1** | **0** | |  |
| 1. **Manual removal of placenta** | **1** | **0** | |  |
| 1. **Special delivery care practices for preventing mother-to-child transmission (PMTCT) of HIV/AIDS** | **1** | **0** | |  |
| 1. **Assisted vaginal delivery (apply vacuum extractor/ventouse or forceps)** | **1** | **0** | |  |
| 1. **Resuscitate a newborn with bag and mask** | **1** | **0** | |  |
| 1. **Maternal death or near miss reviews/audits** | **1** | **0** | |  |
| 1. **Quality improvement approaches such as standards based management** | **1** | **0** | |  |
| **H114: In your current position, and as a part of your work for this facility, do you personally provide care for newborns?** | **1** | **0** | | **No→H118** |
| **H115: How many years in total have you provided such services? Service may have been here or in another facility *(Observer: enter 00 if less than 1 year of service)*** | **\|___\|___\|** | | |  |
| **H116: During the past 2 years have you received any preservice or in-service training on subjects related to newborn care?** | **1** | **0** | | **No→H118** |
| **H117: In the past 2 years, did you receive any training on the following topics *(read each answer aloud)*:** | **1** | **0** | |  |
| 1. **Essential newborn care (e.g., cord care, warming, early and exclusive breastfeeding)** | **1** | **0** | |  |
| 1. **Resuscitation of newbornsnot crying or breathing at birth** | **1** | **0** | | **No→H118** |
| **2a)Did you receive this newborn resuscitation training as part of the Helping Babies Breathe (HBB) Initiative?** | **1** | **0** | | **DK** |
| **2b: Did you ever have the opportunity to practice resuscitating a newborn using a newborn anatomic model/doll (NeoNatalie) after you were trained?**  **2c: Have you had the opportunity to practice resuscitating a newborn using a newborn anatomic model/doll (NeoNatalie) in the last 3 months?** | **1**  **1** | **0**  **0** | | **No→H118** |
| **WORKING CONDITIONS IN FACILITY** |  |  | |  |
| ***Now I would like to ask you some questions about supervision you have personally received. This supervision may have been from a supervisor either in this facility, or from outside the facility.*** | | | | |
| **H118: Do you receive technical support or supervision in your work at this facility?** | **1** | **0** | | **No→H121** |
| **H119: When is the most recent time you were supervised?** | **Last 3 months**  **Last 6 months**  **More than 6 months ago** | | | **A**  **B**  **C** |
| **H120: The last time you were personally supervised, did your supervisor do any of the following *(read each aloud)*:** |  |  | |  |
| 1. **Check your records or reports** | **1** | **0** | |  |
| 1. **Observe your work** | **1** | **0** | |  |
| 1. **Give you verbal feedback about how you were doing your job** | **1** | **0** | |  |
| 1. **Provide any written comment about how you were doing your job** | **1** | **0** | |  |
| 1. **Provide updates on administrative or technical issues related to your work** | **1** | **0** | |  |
| 1. **Discuss problems you have encountered** | **1** | **0** | |  |
| 1. **Participate in quality of care improvement activities** | **1** | **0** | |  |
| 1. **Observe you performing newborn resuscitation with a newborn anatomic model/doll (NeoNatalie)** | **1** | **0** | |  |

| *For question H121, do not read the answer choices aloud. If you are not sure whether an answer given by health worker matches that listed, probe for more detail. If they give an answer that is not listed, move on to their next answer. use the probe to encourage health worker to give 3 answers. If they cannot give an answer, or give only answers that do not appear in list, circle don’t know.* | | | |
| --- | --- | --- | --- |
| **H121: Among the various things related to your working situation that you would like to see improved, can you tell me the three that you think would most improve your ability to provide good quality of care services? (PROBE: Anything else?)** | **Code** |  |  |
| **More support from supervisor** | **A** |  |  |
| **More knowledge/ updates / training** | **B** |  |  |
| **More supplies/drugs** | **C** |  |  |
| **Better quality equipment** | **D** |  |  |
| **Less workload (more staff)** | **E** |  |  |
| **Better working hours / flexible times** | **F** |  |  |
| **More incentives (salary, promotion, holidays, transportation)** | **G** |  |  |
| **Increased security** | **H** |  |  |
| **Better facility infrastructure** | **I** |  |  |
| **More autonomy / independence** | **J** |  |  |
| **Emotional support for staff (verbal encouragement)** | **K** |  |  |
| **More job aids / guidelines / standards** | **L** |  |  |
| **Don't know / None of these** | **Z** |  |  |
| ***END OF SECTION 1*** | | | |
|  | | | |

| Section 2: Maternal Health Knowledge Questions | | | | |
| --- | --- | --- | --- | --- |
| ***For the following questions, read the question aloud to the health worker. Do not read the answer choices aloud. If you are not sure whether an answer given by health worker matches that listed, probe for more detail. If they give an answer that is not listed, move on to their next answer. use the probe to encourage health worker to give as many answers as they can think of. If they cannot give an answer, or give only answers that do not appear in list, circle don’t know.*** | | | | |
| ***READ ALOUD:* Please answer the following questions on maternal health to the best of your knowledge. Most of the questions I ask you will require multiple responses from you. Assume all needed supplies, medications, and equipment are available. When thinking about your answers, you should include actions or interventions that could be done at your facility and at a referral facility. I will probe sometimes to help you remember some more information. Please provide all responses that come to mind.** | | | | |
| **Question** |  | **Code** |  |  |
| **H200: What actions during labor and delivery would you take in an HIV+ woman to prevent/ reduce mother-to-child transmission of the virus?** | |  |  |  |
|  | **PMTCT counseling** | **A** |  |  |
| **(PROBE: Any other actions or interventions?)** | **Provide ARV prophylaxis to woman in early labor** | **B** |  |  |
|  | **Wipe nose, mouth, eyes of newborn with gauze, avoid suction** | **C** |  |  |
|  | **No routine episiotomy** | **D** |  |  |
|  | **Minimize instrument delivery** | **E** |  |  |
|  | **Hibitane vaginal cleansing** | **F** |  |  |
|  | **Minimize vaginal exam** | **G** |  |  |
|  | **Minimize artificial rupture of membranes** | **H** |  |  |
|  | **Avoid milking cord/ immediate clamp cord** | **I** |  |  |
|  | **Appropriate use of partograph** | **J** |  |  |
|  | **Active management of 3rd stage labor** | **K** |  |  |
|  | **Provide ARV prophylaxis to infant** | **L** |  |  |
|  | **Don’t know** | **Z** |  |  |
| **H201: What are the key steps for performing active management of the third stage of labor?** | |  |  |  |
|  | **Administration of a uterotonic immediately/ within 1 minute of delivery** | **A** |  |  |
| **(PROBE: if health worker mentions uterotonic, ask when should uterotonic be given?)** | **Administration of a uterotonic with delivery of anterior shoulder** | **B** |  |  |
|  | **Administration of a uterotonic after delivery of placenta** | **C** |  |  |
|  | **Controlled cord traction** | **D** |  |  |
|  | **Uterine massage** | **E** |  |  |
|  | **Don't know** | **Z** |  |  |
| ***End of Section 2*** | | | | |

| Section 3: Newborn Health Knowledge Questions | | | | |
| --- | --- | --- | --- | --- |
| ***For the following questions, read the question aloud to the health worker. Do not read the answer choices aloud. If you are not sure whether an answer given by health worker matches that listed, probe for more detail. If they give an answer that is not listed, move on to their next answer. use the probe to encourage health worker to give as many answers as they can think of. If they cannot give an answer, or give only answers that do not appear in list, circle don’t know.*** | | | | |
| ***READ ALOUD:*Please answer the following questions on newborn health to the best of your knowledge. Most of the questions I will be asking you will require multiple responses from you. Assume all needed supplies, medications, and equipment are available. When thinking about your answers, you should include actions or interventions that could be done at your facility and at a referral facility. I will probe sometimes to help you remember some more information. Please provide all responses that come to mind.** | | | | |
| **Question** |  | **Code** |  |  |
| **H301: What basic equipment and supplies must be available to ensure the baby receives appropriate immediate care after birth?** | |  |  |  |
|  | **2 dry warm towels or cloths** | **A** |  |  |
| **(PROBE: Anything else?)** | **Sterile blade or scissors** | **B** |  |  |
|  | **Sterile or disposable cord ties/ clamps** | **C** |  |  |
|  | **Cap for baby** | **D** |  |  |
|  | **Source of warmth: heating lamp or incubator** | **E** |  |  |
|  | **Self-inflating ventilation bag** | **F** |  |  |
|  | **Newborn face mask size 1** | **G** |  |  |
|  | **Newborn face mask size 0** | **H** |  |  |
|  | **Mucus extractor/ suction/ bulb syringe** | **I** |  |  |
|  | **Flat surface** | **J** |  |  |
|  | **Clock or watch with seconds** | **K** |  |  |
|  | **Don't know** | **Z** |  |  |
| **H302: Please tell me, when a baby is delivered and there is no complication, what care is important to give them immediately after birth and in the first hour?** | |  |  |  |
|  | **Wipe face after birth of head** | **A** |  |  |
| **(PROBE: Anything else?)** | **Ensure baby was breathing/ crying** | **B** |  |  |
|  | **Provide thermal protection: placeskin to skin with mother** | **C** |  |  |
|  | **Provide thermal protection: wrap baby in a towel/cloth** | **M** |  |  |
|  | **Bathe newborn shortly after birth** | **D** |  |  |
|  | **Suction newborn with bulb** | **E** |  |  |
|  | **Ensure mother initiates breast feeding within 1 hour** | **F** |  |  |
|  | **Assess/examine newborn within 1 hour** | **G** |  |  |
|  | **Weigh newborn** | **H** |  |  |
|  | **Provide eye prophylaxis /antibiotic ointment** | **I** |  |  |
|  | **Give prelacteal feed/ water** | **J** |  |  |
|  | **Cut cord with sterile blade/scissors** | **K** |  |  |
|  | **Apply antiseptic or other material to cord stump** | **L** |  |  |
|  | **Don't know** | **Z** |  |  |

| H303: Can you please tell me the signs and symptoms of severe infection (sepsis) in a newborn? | |  |  |  |
| --- | --- | --- | --- | --- |
|  | **Poor/ no breastfeeding** | **A** |  |  |
| **(PROBE: Any other signs or symptoms?)** | **Restlessness/irritability** | **B** |  |  |
|  | **Breathing difficulties** | **C** |  |  |
|  | **Hypothermia** | **D** |  |  |
|  | **Hyperthermia** | **E** |  |  |
|  | **Breathing rating >60/minute** | **F** |  |  |
|  | **Convulsions** | **G** |  |  |
|  | **Pus/ redness around umbilicus** | **H** |  |  |
|  | **Abscess on any part of body** | **I** |  |  |
|  | **Skin pustules** | **J** |  |  |
|  | **Lethargy/ no movement (conscious)** | **K** |  |  |
|  | **Unconscious** | **L** |  |  |
|  | **Don't know** | **Z** |  |  |
| ***End of interview. Move on to clinical simulations of newborn resuscitation.*** | | | | |
