# Supplementary material for: Health facility service availability and readiness for intrapartum and immediate postpartum care in Malawi: A cross-sectional survey
Source: PLoS One. 2017 Mar 16;12(3):e0172492. doi: 10.1371/journal.pone.0172492 (PMC5354363; doi:10.1371/journal.pone.0172492)
Supplement: S1 Data — (ZIP) [file pone.0172492.s006.zip › Malawi HBB Eval Tools 5_August 18 2012_FINAL.docx]

Q12

| Cover Page | | | |
| --- | --- | --- | --- |
| Q1: Facility name |  | **Q2: Facility code** | \| **C/I** \|  \|  \|  \| \| --- \| --- \| --- \| --- \| |
| \|  \|  \| \| --- \| --- \|   Q3: Observer code |  | **Q4: Today’s date**  **(day/month/year)**   \| **D** \| **D** \| **M** \| **M** \| **Y** \| **Y** \| \| --- \| --- \| --- \| --- \| --- \| --- \| |  |
| *Before observing the consultation, make sure to obtain permission from both the service provider and the client. Also make sure that the provider knows that you are not there to evaluate him or her, and that you are not an “expert” to be consulted during the session.*  *Read The consent script to health worker located on separate consent form*  Q5: *Ask health worker* Do I have your permission to be present at this consultation?  Yes, consent is signed → go to Q6  No, consent is not signed → observation of this health worker must END; if available, approach another health worker for participation. | | | |
| Q6: Health worker line number (from staff listing – Column 1 of Tool 1A) | \|  \|  \| \| --- \| --- \| | **Q7: Sex of health worker** |  |
|  |  |  | **Male 1** |
|  |  |  | **Female 2** |
| Q8: Health worker category |  |  |  |
| Obstetrician/Gynaecologist | **1** |  |  |
| Doctor | **2** |  |  |
| Medical Assistant | **3** |  |  |
| Clinical Officer | **4** |  |  |
| Registered Midwife | **5** |  |  |
| Enrolled Nurse Midwife | **6** |  |  |
| Nurse Midwife Technician | **7** |  |  |
| Student | **8** |  |  |
| Other | **9** |  |  |
| *Read the consent script to client. If client is incapacitated, next of kin or family friend accompanying client may give consent. Consent for client cannot be given by health worker or facility in-charge. Client or proxy consent must be obtained prior to start of observation.*  Q9: *Ask client* Do I have your permission to be present while you are receiving services today?  Yes, consent is signed → go to Q10  No, consent is not signed → observation of this client must END; if available, approach another client for participation. | | | |
| Q10: Who gave consent | **Client 1** |  | |
|  | **Next of kin/family friend 2** |  |  |
| Q11: Client code   \|  \|  \|  \| \| --- \| --- \| --- \| |  | ***Start client code at 001 for the first client observed at a given facility.*** | |
| Q12: Client initials or other code |  | ***Write client initials or code in box at top right of cover first page (marked Q12) to help identify this client’s case when observing multiple cases*** | |
| Q13: Record time the observation started (*Observer:* Please use 24 hr clock) | \|  \|  \|  \|  \| \| --- \| --- \| --- \| --- \| |  |  |
| *Go to Section 3 to begin the observation* | | | |
|  | | | |

| Section 3: Continuous Observation of Second & Third Stage of Labor (TOOL 5) | | | | |
| --- | --- | --- | --- | --- |
| **Question** | **Yes** | **No** | **DK** | **Go to** |
| ***Record whether the provider carried out the following steps and/or examinations: (some of the following steps may be performed simultaneously or by more than one provider).*** | | | | |
| **PREPARATION FOR DELIVERY** |  |  |  |  |
| **Q301: Washes his/her hands with soap and water or uses antiseptic before any examination of woman *(observer: circle yes if done previously and no contamination)*** | **1** | **0** | **8** |  |
| **Q302: Wears high-level disinfected or sterile surgical gloves *(yes if no contamination)*** | **1** | **0** | **8** |  |
| **Q303: Puts on clean protective clothing in preparation for birth (goggles, gown or apron) *(yes if no contamination)*** | **1** | **0** | **8** |  |
| **Q304: Performs episiotomy** | **1** | **0** |  |  |
| **Q305: Presentation of baby is cephalic (head first)** | **1** | **0** | **8** |  |
| **DELIVERY & UTEROTONIC** |  |  |  |  |
| **Q306: As baby's head is delivered, supports perineum** | **1** | **0** | **8** |  |
| **Q307: Record time of the delivery of the baby**  **(*Observer:* Please use 24 hr clock)** | \|  \|  \|  \|  \| \| --- \| --- \| --- \| --- \| | |  |  |
| **Q308: Checks for another baby prior to giving the uterotonic** | **1** | **0** | **8** |  |
| **Q309: Second baby present? *(observer: circle 1 if multiple babies)*** | **1** | **0** |  |  |
| **Q310: Administers uterotonic?** | **1** | **0** |  | **No → Q317** |
| **Q311: Record time uterotonic given**  **(*Observer:* Please use 24 hr clock)** | \|  \|  \|  \|  \| \| --- \| --- \| --- \| --- \| | |  |  |
| **Q312:Timing of administration of uterotonic** | **Code** |  |  |  |
| **At delivery of anterior shoulder** | **1** |  |  |  |
| **Within 1 min of delivery of baby** | **2** |  |  |  |
| **Within 3 min of delivery of baby** | **3** |  |  |  |
| **More than 3 min after delivery of baby AND before delivery of the placenta** | **4** |  |  |  |
| **More than 3 min of delivery of baby and after delivery of placenta** | **5** |  |  |  |
| **Q313: Which uterotonic given** |  |  |  |  |
| **Oxytocin** | **1** |  |  |  |
| **Ergometrine** | **2** |  |  |  |
| **Syntometrine** | **3** |  |  |  |
| **Misoprostol** | **4** |  |  |  |
| **Q314: Record dose of uterotonic given *(observer: if necessary, ask afterwards)*** | \|  \|  \| \| --- \| --- \| | |  |  |
| **Q315: Units of medication *(observer: if necessary, ask afterwards)*** |  |  |  |  |
| **IU** | **1** |  |  |  |
| **mg** | **2** |  |  |  |
| **mL** | **3** |  |  |  |
| **mcg** | **4** |  |  |  |
| **Q316: Route uterotonic given:** |  |  |  |  |
| **IM** | **1** |  |  |  |
| **IV** | **2** |  |  |  |
| **Oral** | **3** |  |  |  |
| **Other** | **4** |  |  |  |
| **Q317: Record time the cord was clamped**  **(*Observer:* Please use 24 hr clock)** | \|  \|  \|  \|  \| \| --- \| --- \| --- \| --- \| | |  |  |
| **Question** | **Yes** | **No** | **DK** |  |
| **Q318: Applies traction to the cord while applying suprapubic counter traction** | **1** | **0** | **8** |  |
| **Q319: Performs uterine massage immediately following the delivery of the placenta** | **1** | **0** | **8** |  |
| **Q320: Was placenta delivered before administration of uterotonic? *(observer: circle Don’t Know if no uterotonic was given)*** | **1** | **0** | **8** |  |
| **Q321: Assesses completeness of the placenta and membranes** | **1** | **0** | **8** |  |
| **Q322: Assesses for perineal and vaginal lacerations** | **1** | **0** | **8** |  |
| **Q323: *Observer:* Did more than one health worker assist with the birth?** | **1** | **0** |  |  |
| **Q324: *Observer:* Did mother gave birth in lithotomy position (on back)** | **1** | **0** |  |  |
| **Q325: *Observer:* Is a support person (companion) for mother present at birth?** | **1** | **0** |  |  |
| ***End of Section 3*** | | | | |

| Section 4: Immediate Newborn and Postpartum Care (TOOL 5) | | | | |
| --- | --- | --- | --- | --- |
| **Question** | **Yes** | **No** |  | **Go to** |
| ***Record whether the provider carried out the following steps and/or examinations: (some of the following steps may be performed simultaneously or by more than one provider)*** | | | | |
| **IMMEDIATE CARE** |  |  |  |  |
| **Q401: Immediately dries baby with towel** | **1** | **0** | **8** |  |
| **Q402: Discards the wet towel** | **1** | **0** | **8** |  |
| **Q403: Is the baby breathing or crying?** | **1** | **0** |  | **No → Q501** |
| ***If baby is not breathing or crying AT BIRTH, go to resuscitation checklist GO to Q501*** |  |  |  |  |
| **Q404: Places baby on mother’s abdomen “skin to skin”** | **1** | **0** | **8** |  |
| **Q405: Covers baby with dry towel** | **1** | **0** | **8** |  |
| **Q406: If not placed skin to skin, wraps baby in dry towel** | **1** | **0** | **8** |  |
| **Q407: Ties or clamps cord when pulsations stop, or by 2-3 minutes after birth (not immediately after birth)** | **1** | **0** | **8** |  |
| **Q408: Cuts cord with clean blade or clean scissors** | **1** | **0** | **8** |  |
| **Q409: Observer: Is a support person (companion) for mother present?** | **1** | **0** |  |  |
| **HEALTH CHECK** |  |  |  |  |
| **Q410: Checks baby's temperature 15 minutes after birth** | **1** | **0** | **8** |  |
| **Q411: Checks baby's skin color 15 minutes after birth** | **1** | **0** | **8** |  |
| **Q412: Takes mother's vital signs 15 minutes after birth** | **1** | **0** | **8** |  |
| **Q413: Palpates uterus 15 minutes after delivery of placenta** | **1** | **0** | **8** |  |
| **FIRST HOUR AFTER BIRTH** |  |  |  |  |
| **Q414: Mother and newborn kept in same room after delivery (rooming-in)** | **1** | **0** | **8** |  |
| **Q415: Baby bathed within the first hour after birth** | **1** | **0** | **8** |  |
| **Q416: Baby kept skin to skin with mother for the first hour after birth** | **1** | **0** | **8** |  |
| **Q417: Breastfeeding initiated within the first 30 minutes after birth** | **1** | **0** | **8** |  |
| **Q417a: Breastfeeding initiated within the first hour after birth** | **1** | **0** | **8** |  |
| **Q418: Provides tetracycline eye ointment prophylaxis** | **1** | **0** | **8** |  |
| **Q419: Administers Vitamin K to newborn** | **1** | **0** | **8** |  |
| **Q420: Is the mother HIV positive? *(observer: listen and record answer; circle Don’t Know if status is unknown or is not discussed)*** | **1** | **0** | **8** | **No/DK → Q422** |
| **Q421: Administers ARVs to newborn** | **1** | **0** | **8** |  |
| **Q422: Administers antibiotics to mother postpartum** | **1** | **0** | **8** | **No/DK → Q425** |
| **Q423: Why were antibiotics administered?** | **Code** |  |  |  |
| **Treatment for chorioamnionitis** | **1** |  |  |  |
| **Routine/prophylactic** | **4** |  |  |  |
| **Third stage/postpartum procedure** | **5** |  |  |  |
| **Don't know** | **8** |  |  |  |
| **Q424: Which antibiotic was administered? (CIRCLE ALL THAT APPLY)** |  |  |  |  |
| **Penicillin** | **A** |  |  |  |
| **Ampicillin** | **B** |  |  |  |
| **Gentamicin** | **C** |  |  |  |
| **Metronidazole** | **D** |  |  |  |
| **Cephalosporin** | **E** |  |  |  |
| **Other** | **X** |  |  |  |
| **Don’t know** | **Z** |  |  |  |
| **CLEAN-UP AFTER BIRTH** |  |  |  |  |
| ***Record whether the provider carried out the following steps and/or examinations: (some of the following steps may be performed simultaneously or by more than one provider)*** | | | | |
| **Question** | **Yes** | **No** | **DK** | **Go to** |
| **Q425: Disposes of all sharps in a puncture-proof container immediately after use** | **1** | **0** | **8** |  |
| **Q426: Decontaminates all reusable instruments in 0.5% chlorine solution** | **1** | **0** | **8** |  |
| **Q427: Sterilizes or uses high-level disinfection for all reusable instruments** | **1** | **0** | **8** |  |
| **Q428: Disposes of all contaminated waste in leak-proof containers** | **1** | **0** | **8** |  |
| **Q429: Removes apron and wipe with chlorine solution** | **1** | **0** | **8** |  |
| **Q430: Washes his/her hands with soap and water or uses antiseptic** | **1** | **0** | **8** |  |
|  |  |  |  |  |
| **CLEAN-UP AFTER NEWBORN RESUSCITATION** | **YES** | **NO** | **DK** |  |
| **Q431: Was there a newborn resuscitation?** | **1** | **0** |  | **No → Q601** |
| **Q432: Disposes of disposable suction catheters and mucus extractors in a leak-proof container or plastic bag** | **1** | **0** | **8** |  |
| **Q433: Takes the bag and mask apart and inspects for cracks and tears** | **1** | **0** | **8** |  |
| **Q434: Decontaminates the bag and mask in 0.5% chlorine solution** | **1** | **0** | **8** |  |
| **Q435: Sterilizes or uses high-level disinfection for bag, valve and mask** | **1** | **0** | **8** |  |
| **Q436: Decontaminates reusable suction devices in 0.5% chlorine solution** | **1** | **0** | **8** |  |
| **Q437: Sterilizes or uses high-level disinfection for reusable suction devices** | **1** | **0** | **8** |  |
| **Q438: Washes his/her hands with soap and water or uses antiseptic** | **1** | **0** | **8** |  |
| **Record time L&D observation ended**  **(24 hr clock)** | \|  \|  \|  \|  \| \| --- \| --- \| --- \| --- \| | |  |  |
| ***Remember to thank client and provider for their participation in the study*** | | | | |
|  | | | | |

| Section 5: Checklist for Newborn Resuscitation (TOOL 5) | | | | |
| --- | --- | --- | --- | --- |
| **Question** | **Yes** | **No** | **DK** | **Go to** |
| ***Record whether the provider carried out the following steps and/or examinations: (some of the following steps may be performed simultaneously or by more than one provider)*** | | | | |
| **Q501: Record time resuscitation started (*Observer:* Please use 24 hr clock)** | \|  \|  \|  \|  \| \| --- \| --- \| --- \| --- \| | |  |  |
| **Q502: Clears the airway by suctioning the mouth first and then the nose** | **1** | **0** | **8** |  |
| **Q503: Stimulates baby with back rubbing** | **1** | **0** | **8** |  |
| **Q504: *OBSERVER:* does newborn starts to breathe or cry spontaneously?** | **1** | **0** |  | **Yes→Q531** |
| **Q506: Ties or clamps cord immediately** | **1** | **0** | **8** |  |
| **Q507: Cuts cord with clean blade or clean scissors** | **1** | **0** | **8** |  |
| **Q508: Places the newborn on his/her back on a clean, warm surface or towel** | **1** | **0** | **8** |  |
| **Q509: Places the head in a slightly extended position to open the airway** | **1** | **0** | **8** |  |
| **Q510: Tells the woman (and her support person) what is going to be done** | **1** | **0** | **8** |  |
| **Q511: Listens woman and provides support and reassurance** | **1** | **0** | **8** |  |
| **Q512: Checks mouth, back of throat and nose for secretions, and clears if necessary** | **1** | **0** | **8** |  |
| **Q513: Places the correct-sized mask on the newborn’s face so that it covers the chin, mouth and nose (but not eyes)** | **1** | **0** | **8** |  |
| **Q514: Checks the seal by ventilating two times and observing the rise of the chest** | **1** | **0** | **8** |  |
| **Q515: *OBSERVER:* is newborn’s chest rising in response to ventilation?** | **1** | **0** |  | **Yes→Q524** |
| **Q515a:Calls for help** | **1** | **0** | **8** |  |
| **Q516: Checks the position of the newborn’s head to make sure that the neck is in a slightly extended position (not blocking the airway)** | **1** | **0** | **8** |  |
| **Q517: Checks mouth, back of throat and nose for secretions, and clears if necessary** | **1** | **0** | **8** |  |
| **Q518: Checks the seal by ventilating two times and observing the rise of the chest** | **1** | **0** | **8** |  |
| **Q519: *OBSERVER:* is newborn’s chest rising in response to ventilation?** | **1** | **0** |  | **Yes→Q524** |
| **Q520: Checks the position of the newborn’s head again to make sure that the neck is in slightly extended position** | **1** | **0** | **8** |  |
| **Q521: Repeats suction of mouth and nose to clear secretions, if necessary** | **1** | **0** | **8** |  |
| **Q522: Checks the seal by ventilating two times and observing the rise of the chest** | **1** | **0** | **8** |  |
| **Q523: *OBSERVER:* is newborn’s chest rising in response to ventilation?** | **1** | **0** |  | **Yes→Q524** |
| ***If newborn's chest is not rising after two attempts to readjust, observer should call for supervisor to intervene. If a health worker competent in resuscitation is not available, observer may choose to intervene.*** | | | | |
| **Q524: Ventilates at a rate of 30 to 50 breaths/minute** | **1** | **0** | **8** |  |
| **Q525: Conducts assessment of newborn breathing after 1 minute of ventilation** | **1** | **0** |  | **No→Q527** |
| **Q526: Condition of newborn at assessment** | **Code** |  |  |  |
| **Respiration rate 30-50 breaths/minute and no chest indrawing** | **1** |  |  | **→Q531** |
| **Respiration rate <30 breaths/minute with severe indrawing** | **2** |  |  |  |
| **No spontaneous breathing** | **3** |  |  |  |
| **Q526a: Checks for heart rate** | **1** | **0** | **8** |  |
|  | **Yes** | **No** | **DK** | **Go to** |
| **Q527: Continues Ventilation and baby cries before 10 minutes** | **1** | **0** |  | **Yes→Q529** |
| **Q528: Conducts assessment of newborn breathing after prolonged ventilation (10 minutes)** | **1** | **0** |  | **No→Q530** |
| **Q529: Condition of newborn at assessment** | **Code** |  |  |  |
| **Respiration rate 30-50 breaths/minute and no chest indrawing** | **1** |  |  | **→Q531** |
| **Respiration rate <30 breaths/minute with severe indrawing** | **2** |  |  |  |
| **No spontaneous breathing** | **3** |  |  |  |
|  | **Yes** | **No** | **DK** | **Go to** |
| **Q530: Continues Ventilation** | **1** | **0** |  |  |
| **Q531: Record time that resuscitation actions ended (or time of death if baby died)**  **(*Observer:* Please use 24 hr clock)** | \|  \|  \|  \|  \| \| --- \| --- \| --- \| --- \| | |  |  |
| **Q532: Was the resuscitation successful? *(observer: circle No if newborn died)*** | **1** | **0** |  |  |
| **Q533: Arranges transfer to special care either in facility or to outside facility** | **1** | **0** | **8** |  |
| **Q534: Explains to the mother (and her support person if available) what happened** | **1** | **0** | **8** |  |
| **Q535: Listens to mother and responds attentively to her questions and concerns** | **1** | **0** | **8** |  |
| **Q536: Observer: Did you call for help or intervene during the resuscitation to save the life of newborn?** | **1** | **0** |  |  |
|  |  |  |  |  |
| **Q537: *Please comment on the quality of care provided:***  ***Was mother treated respectfully? Informed of procedures to her baby? Was the situation chaotic or calm? Were there any major delays in needed treatment? If so, for what drugs/procedures and why? Were multiple health workers involved? Who?* *If newborn did not survive, describe the circumstances. Was the mother counseled about the death of newborn?*** | | | | |
| **OBSERVER: PLEASE RETURN TO Q410 (CLEAN-UP AFTER BIRTH)** | | | | |

| Section 6: Outcome & Review of Documentation | | | | |
| --- | --- | --- | --- | --- |
| **Question** | **Code** |  |  |  |
| ***Complete this section for all clients*** | | | | |
| **CONDITION OF MOTHER & NEWBORN AT END OF OBSERVATION** |  |  |  |  |
| ***Record the status of mother and newborn at the end of first hour after birth.*** | | | | |
| **Q601: Record outcome for the mother** |  |  |  |  |
| **Goes to recuperation ward** | **1** |  |  |  |
| **Referred to specialist, same facility** | **2** |  |  |  |
| **Goes to surgery, same facility** | **3** |  |  |  |
| **Referred, other facility** | **4** |  |  |  |
| **Death of mother** | **5** |  |  |  |
| **Don't know** | **8** |  |  |  |
| **Q602: Record outcome for the newborn or fetus** |  |  |  |  |
| **Goes to normal nursery** | **1** |  |  |  |
| **Referred to specialist, same facility** | **2** |  |  |  |
| **Referred, other facility** | **3** |  |  |  |
| **Goes to ward with mother** | **4** |  |  |  |
| **Newborn death** | **5** |  |  |  |
| **Fresh stillbirth** | **6** |  |  |  |
| **Macerated stillbirth** | **7** |  |  |  |
| **Don't know** | **8** |  |  |  |
| **POTENTIALLY HARMFUL PRACTICES** |  |  |  |  |
| **Q603: Did you see any of the following harmful or inappropriate practices by health workers that are never indicated (CIRCLE ALL THAT APPLY)** |  |  |  |  |
| **Use of enema** | **A** |  |  |  |
| **Public shaving** | **B** |  |  |  |
| **Apply fundal pressure to hasten delivery of baby or placenta** | **C** |  |  |  |
| **Lavage of uterus after delivery** | **D** |  |  |  |
| **Slap newborn** | **E** |  |  |  |
| **Hold newborn upside down** | **F** |  |  |  |
| **Milking the newborn's chest** | **G** |  |  |  |
| **Excessive stretching of the perineum** | **H** |  |  |  |
| **Shout, insult or threaten the woman during labor or after** | **I** |  |  |  |
| **Slap, hit or pinch the woman during labor or after** | **J** |  |  |  |
| **None of the above** | **Y** |  |  |  |
| **Q604: Did you see any of the following practices done without an appropriate indication (CIRCLE ALL THAT APPLY)** |  |  |  |  |
| **Manual exploration of the uterus after delivery** | **A** |  |  |  |
| **Use of episiotomy** | **B** |  |  |  |
| **Aspiration of newborn mouth and nose as soon as head is born** | **C** |  |  |  |
| **Restrict food and fluids in labor** | **D** |  |  |  |
| **None of the above** | **Y** |  |  |  |
| **REVIEW PARTOGRAPH AND/OR CHART FOR COMPLETENESS** |  |  |  |  |
| **Question** | **Yes** | **No** | **DK** | **Go to** |
| **Q605: Was there a newborn resuscitation? *(observer: check answer to Q500)*** | **1** | **0** |  | **No → Q611** |
| ***Examine chart to determine whether the health worker recorded the following information:*** | | | | |
| **Q606: Condition of the newborn at birth** | **1** | **0** | **8** |  |
| **Q607: Procedures necessary to initiate breathing** | **1** | **0** | **8** |  |
| **Q608: Time from birth to initiation of spontaneous breathing or time of death if unsuccessful** | **1** | **0** | **8** |  |
| **Q609: Any clinical observations during resuscitation, including baby vital signs** | **1** | **0** | **8** |  |
| **Q610: Final outcome of resuscitation measures** | **1** | **0** | **8** |  |
| ***Examine partograph if available*** | | | | |
| **Q611: Partograph used to monitor labor** | **1** | **0** |  | **No → Q630** |
| **Q612: Which partograph used** | **Code** |  |  |  |
| **Old WHO partograph (latent phase)** | **1** |  |  |  |
| **New WHO partograph (at 4cm dilatation)** | **2** |  |  |  |
| **Other partograph** | **3** |  |  |  |
| **Question** | **Yes** | **No** | **DK** | **Go to** |
| **Q613: Initiated use of partograph at the appropriate time according to partograph used (New WHO partograph starts at 4 cm; old version starts at 3 cm)** | **1** | **0** | **8** |  |
|  | | | | |
| ***Examine partograph to determine whether the health worker recorded the following information while the woman was in active labor:*** | | | | |
| **Q614: Fetal heart rate plotted at least every half hour** | **1** | **0** | **8** |  |
| **Q615: Cervical dilatation plotted at least every four hours** | **1** | **0** | **8** |  |
| **Q616: Descent of head plotted at least every four hours** | **1** | **0** | **8** |  |
| **Q617: Frequency and duration of contractions plotted at least every half hour** | **1** | **0** | **8** |  |
| **Q618: Maternal pulse plotted at least every half hour** | **1** | **0** | **8** |  |
| **Q619: BP recorded at least every four hours** | **1** | **0** | **8** |  |
| **Q620: Temperature recorded at least every two hours** | **1** | **0** | **8** |  |
| **Q621: OBSERVER: Did you see provider fill out partograph after delivery (with information that should be entered during labor)? (circle Don’t Know if partograph use was not observed)** | **1** | **0** | **8** |  |
| ***Examine partograph to determine whether the health worker recorded the following information about the delivery*** | | | | |
| **Q622: Birth time** | **1** | **0** | **8** |  |
| **Q623: Delivery method** | **1** | **0** | **8** |  |
| **Q624: Birth weight** | **1** | **0** | **8** |  |
| **DATA EXTRACTION FROM PARTOGRAPH AND/OR CHART** |  |  |  |  |
| **Q625: Was action line on partograph reached?** | **1** | **0** | **8** | **No/DK → Q630** |
| **Q626: Record time action line was reached**  **(*Observer:* Please use 24 hr clock)** | \|  \|  \|  \|  \| \| --- \| --- \| --- \| --- \| | |  |  |
| **Q627: If action line reached on partograph, was any definitive action taken?** | **1** | **0** | **8** | **No/DK → Q630** |
| **Q628: Record time action was taken *(observer: enter 99:99 if unknown)***  **(*Observer:* Please use 24 hr clock)** | \|  \|  \|  \|  \| \| --- \| --- \| --- \| --- \| | |  |  |
| **Q629: What definitive action was taken:** | **Code** |  |  |  |
| **Consult with specialist** | **1** |  |  |  |
| **Refer to other facility for specialist** | **2** |  |  |  |
| **Prepare for assisted delivery** | **3** |  |  |  |
| **Prepare for c-section** | **4** |  |  |  |
| **Other (specify_____________)** | **6** |  |  |  |
| ***For the following questions: Examine partograph and/or chart to determine the following information. If the information is not in the chart or partograph, but the observer knows the information or previously recorded the information in another section, he or she should fill in their own answer. If the information in the chart or partograph differ from observer's information, use observer's information.*** | | | | |
| **Q630: Record age of woman** | \|  \|  \| \| --- \| --- \| |  |  |  |
| **Q631: Record the gravidity of the woman** | \|  \|  \| \| --- \| --- \| |  |  |  |
| **Q632: Record the parity of the woman prior to this delivery** | \|  \|  \| \| --- \| --- \| |  |  |  |
| **Q633: Time of admission to labor ward *(observer: enter 99:99 if unknown)***  **(*Observer:* Please use 24 hr clock)** | \|  \|  \|  \|  \| \| --- \| --- \| --- \| --- \| | |  |  |
| **Q634: Centimeters dilated upon admission to labor ward *(observer: enter 99 if unknown)*** | \|  \|  \| \| --- \| --- \| |  |  |  |
| **Q635: Time membranes ruptured *(observer: enter 99:99 if unknown)***  **(*Observer:* Please use 24 hr clock)** | \|  \|  \|  \|  \| \| --- \| --- \| --- \| --- \| | |  |  |
| **Q636: How did the membranes rupture?** | **Code** |  |  |  |
| **Spontaneous** | **1** |  |  |  |
| **Artificial** | **2** |  |  |  |
| **Don't know** | **8** |  |  |  |
| **Q637: Type of delivery** |  |  |  |  |
| **Spontaneous vaginal** | **1** |  |  |  |
| **Assisted (instrumented)** | **2** |  |  |  |
| **Caesarean** | **3** |  |  |  |
| **Don't know** | **8** |  |  |  |
| **Q638: Time of birth *(observer: enter 99:99 if unknown)***  **(*Observer:* Please use 24 hr clock)** | \|  \|  \|  \|  \| \| --- \| --- \| --- \| --- \| | |  |  |
| **Q639: Birth weight in grams *(observer: enter 9999 if unknown)*** | \|  \|  \|  \|  \| \| --- \| --- \| --- \| --- \| | |  |  |
| **Q640: Record gestational age at birth in weeks *(observer: enter 99 if unknown)*** | \|  \|  \| \| --- \| --- \| |  |  |  |
| **Question** | **Yes** | **No** | **DK** | **Go to** |
| **Q641: Was she diagnosed with severe PE/E?** | **1** | **0** | **8** | **No→ Q643** |
| **Q642: Was baby delivered within 24 hours of PE/E diagnosis?** | **1** | **0** | **8** |  |
| **Q643: Did the mother have blood loss more than 500mL?** | **1** | **0** | **8** | **No→ Q645** |
| **Q644: Was she diagnosed with postpartum hemorrhage?** | **1** | **0** | **8** |  |
| **Q645: Did the mother develop a fever of 38° C or higher during labor?** | **1** | **0** | **8** | **No→ Q647** |
| **Q646: Was she diagnosed with chorioamnionitis during labor?** | **1** | **0** | **8** |  |
| **Q647: Were antibiotics administered to mother at any time?** | **1** | **0** | **8** | **No/DK → Q651** |
| **Q648: When were antibiotics administered? (CIRCLE ALL THAT APPLY)** | **Code** |  |  |  |
| **1st stage** | **A** |  |  |  |
| **2nd stage** | **B** |  |  |  |
| **3rd stage** | **C** |  |  |  |
| **Postpartum** | **D** |  |  |  |
| **Q649: Why were antibiotics administered? (CIRCLE ALL THAT APPLY)** |  |  |  |  |
| **Treatment for chorioamnionitis** | **A** |  |  |  |
| **After prelabor rupture of membranes** | **B** |  |  |  |
| **Preparation for C-section** | **C** |  |  |  |
| **Routine/prophylactic** | **D** |  |  |  |
| **Third stage/postpartum procedure** | **E** |  |  |  |
| **Don't know** | **Z** |  |  |  |
| **Q650: Which antibiotic was administered? (CIRCLE ALL THAT APPLY)** |  |  |  |  |
| **Penicillin** | **A** |  |  |  |
| **Ampicillin** | **B** |  |  |  |
| **Gentamicin** | **C** |  |  |  |
| **Metronidazole** | **D** |  |  |  |
| **Cephalosporin** | **E** |  |  |  |
| **Other** | **X** |  |  |  |
| **Don't know** | **Z** |  |  |  |
| **Question** | **Yes** | **No** | **DK** | **Go to** |
| **Q651: Is mother HIV positive? *(observer: circle Don’t Know if status is unknown or was not discussed)*** | **1** | **0** | **8** | **No/DK → Q654** |
| **Q652: Was newborn given ARV(s)?** | **1** | **0** | **8** | **No/DK → Q654** |
| **Q653: Record type of ARV(s) given to newborn** | **Code** |  |  |  |
| **NVP** | **1** |  |  |  |
| **AZT** | **2** |  |  |  |
| **3TC** | **3** |  |  |  |
| **Don’t know** | **8** |  |  |  |
| **Q654: *Please comment on the quality of care provided:***  ***Was mother treated respectfully? Informed of procedures to herself and her baby? Was the situation chaotic or calm? Were there any major delays in needed treatment? If so, for what drugs/procedures and why? Were multiple health workers involved? Who? If maternal or newborn/fetal death occurred, describe the circumstances. Was the mother counseled about the death of newborn/fetus?*** | | | | |
