# Supplementary material for: Health facility service availability and readiness for intrapartum and immediate postpartum care in Malawi: A cross-sectional survey
Source: PLoS One. 2017 Mar 16;12(3):e0172492. doi: 10.1371/journal.pone.0172492 (PMC5354363; doi:10.1371/journal.pone.0172492)
Supplement: S1 Data — (ZIP) [file pone.0172492.s006.zip › Malawi HBB Eval_Tool 3_Clinical Simulation_Aug 18 2012 FINAL.docx]

**Tool 3: Clinical case studies for Health Workers**

**Evaluation of a Facility-based scale-up of the HBB Initiative**

| Cover Sheet | | | |
| --- | --- | --- | --- |
| H1: Facility name |  | H2: Facility number | \|__C/I__\|____\|____\|____\| |
| H3: Observer number | \|____\|____\| | H4: Today’s date  DD/MM/YY | \|__D__\|__D__\|__M__\|__M__\|__Y__\|__Y__\| |
| H6: Health worker line number (from staff listing) | \|___\|___\| | H7: Sex of health worker | Male 1  Female 2 |

**-------------------------------------------------------------------------------------------------------------------------------**

**Case Scenario 1**

**Instructions to the Data Collector**:

Hand over the Neonatalie and other equipment to the health worker. If the health worker is not familiar with the NeoNatalie model and resuscitation equipment, identify each piece of equipment and allow the health worker to examine the model thoroughly. Explain how the model works (e.g., the chest will rise when there is a proper seal with the bag and mask, etc.). Then read aloud to the health worker the following instructions:

Read aloud to the health worker the following instructions and the case. Provide prompt where shown in underline. As you observe the worker, tick the boxes “Done” or “not Done” for each activity. Indicate the baby’s response to the health worker’s actions using the neonatal simulator or words if using a mannequin. For example, when the worker evaluates crying, show or say that the baby is not crying.

Data collector: Please read. “I am going to read a role play case. Please listen carefully, and then show me the actions you would take. I will indicate the baby’s response with the stimulator (or in words), but I will provide no other feedback until the end of the case.”

”you are called to assist the delivery of a term baby. There are no complications in the pregnancy. The baby will be born in less than 10 minutes. Introduce yourself and prepare for the birth and care of the baby.”

1. ***Prepares for birth***

|  | **Done (1)** | **Not Done (0)** | |
| --- | --- | --- | --- |
| 1. Identify a helper | ☐ | ☐ |  |
| 1. Makes an emergency plan | ☐ | ☐ |  |
| 1. Prepares the area for delivery | ☐ | ☐ |  |
| 1. Cleans hands and maintains clean technique throughout | ☐ | ☐ |  |
| 1. Prepares an area for ventilation | ☐ | ☐ |  |
| 1. Checks equipment | ☐ | ☐ |  |

**--------------------------------------------------------------------------------------------------------------------------**

1. ***Keeps baby warm***

Prompt for observer: After 5-7 minutes give baby to worker and say, “The amniotic fluid is clear. Show how you will care for the baby.”

| 1. **DRIES THOROUGHLY** | ☐ | ☐ |
| --- | --- | --- |
| 1. Removes wet cloth | ☐ | ☐ |
| 1. Covers baby with dry cloth | ☐ | ☐ |

**--------------------------------------------------------------------------------------------------------------------------**

1. ***Evaluates crying***

Prompt for observer: Show or say the baby is not crying.

| 1. **RECOGNIZES BABY IS NOT CRYING** | ☐ | ☐ |
| --- | --- | --- |

**--------------------------------------------------------------------------------------------------------------------------**

1. ***Clears airway and stimulates breathing***

| 1. **POSITIONS HEAD AND CLEARS AIRWAY** | ☐* | ☐ |
| --- | --- | --- |
| 1. Stimulates breathing by rubbing the back | ☐ | ☐ |

**--------------------------------------------------------------------------------------------------------------------------**

1. ***Evaluates breathing***

Prompt for observer: Show or say the baby is breathing well.

| 1. Recognizes baby is breathing well | ☐ | ☐ |
| --- | --- | --- |
| 1. Clamps or ties and cuts the cord | ☐ | ☐ |
| 1. Position skin-to-skin on mother’s chest | ☐ | ☐ |
| 1. Communicates with mother | ☐ | ☐ |

**--------------------------------------------------------------------------------------------------------------------------**

SCORING:

Please add together the number of activities/ steps that were done and write it in the space below.

Number Done Correctly |____|____|

Data Collector’s code |____|____|

Were the following activities/ steps done? **Yes/ No**

**DRIES THROUGHLY, RECOGNIZES BABY IS NOT CRYING, AND POSITIONS HEAD AND CLEARS AIRWAYS.**

**Case Scenario 2**

***Instructions to the data collector***

Read aloud to the worker the following instructions and the case. Provide prompts where shown in underline. As you observe the worker, tick the boxes “done” or “Not done” for each activity. Indicate to the workers actions using neonatal simulator or words. Note the time between birth and beginning ventilation. Comment on the worker’s performance only at the end of the case.

“I am going to read a role play case. Please listen very carefully, and then show me how you would care for this baby. I will indicate the baby’s response with the stimulator (or in words). I will provide no other feedback until the end of the case.”

“You are called to assist at the birth of 34week (7⅟2 month) gestation baby. You arrive two minutes prior to birth. Introduce yourself and show what you will do.”

|  | **Done (1)** | | | **Not Done (0)** | | |
| --- | --- | --- | --- | --- | --- | --- |
| 1. ***Prepares for a birth*** | | |  | | |  |
| 1. Identifies a helper | | ☐ | | | ☐ | |
| 2. Prepares the area for delivery | | ☐ | | | ☐ | |
| 3. Cleans hands | | ☐ | | | ☐ | |
| 4. Prepares an area for ventilation | | ☐ | | | ☐ | |
| 5. Checks equipment | | ☐ | | | ☐ | |

**--------------------------------------------------------------------------------------------------------------------------**

1. ***Keeps baby warm***

Prompt for observer: After 2 minutes give baby to health worker and say, “The amniotic fluid is clear. Show how you will care for the baby.”

| 1. Dries thoroughly | ☐ | ☐ |
| --- | --- | --- |
| 1. Removes wet cloth | ☐ | ☐ |
| 1. Covers baby with dry cloth | ☐ | ☐ |

**--------------------------------------------------------------------------------------------------------------------------**

1. ***Evaluates crying***

Prompt for observer: Show or say the baby is not crying.

| 1. Recognizes baby is not crying | ☐ | ☐ |
| --- | --- | --- |

**--------------------------------------------------------------------------------------------------------------------------**

1. ***Clears airway and stimulates breathing***

| 1. Positions head | ☐ | ☐ |
| --- | --- | --- |
| 1. Clears airway | ☐ | ☐ |
| 1. Stimulates breathing by rubbing the back | ☐ | ☐ |

**--------------------------------------------------------------------------------------------------------------------------**

1. ***Evaluates breathing***

| 1. **RECOGNIZES BABY IS NOT BREATHING** | ☐* | ☐ |
| --- | --- | --- |

**--------------------------------------------------------------------------------------------------------------------------**

***F. Ventilates with bag and mask***

| 1. Cuts cord | ☐ | ☐ |
| --- | --- | --- |
| 1. Moves to area for ventilation OR ventilates by mother | ☐ | ☐ |
| 3. Starts ventilation within the Golden Minute (at__  seconds) | ☐ | ☐ |
| **4. *VENTILATE AT 40 BREATHES/MINUTE (30-50***  ***ACCEPTABLE)*** | ☐* | ☐ |
| **5. *LOOKS FOR CHEST MOVEMENT*** | ☐* | ☐ |

**--------------------------------------------------------------------------------------------------------------------------**

***G. Evaluate breathing***

Prompt for observer: Show or say the baby is not breathing

| 1. Recognizes baby is not breathing | ☐ | ☐ |
| --- | --- | --- |
| 2. Calls for help | ☐ | ☐ |
| 3. Continues ventilation | ☐ | ☐ |

**--------------------------------------------------------------------------------------------------------------------------**

***H. IMPROVES VENTILATION***

Prompt for observer: Say, “Please show what to do if the chest is not moving with ventilation.” After one or more steps to improve ventilation say, “ The chest is moving now.”

| 1. Head –reposition head | ☐* | ☐ |
| --- | --- | --- |
| 2. Reapplies mask | ☐* | ☐ |
| 3. Mouth- clears secretion, opens mouth slightly | ☐* | ☐ |
| 4. Bag- squeezes bag harder | ☐* | ☐ |

**--------------------------------------------------------------------------------------------------------------------------**

***I. Evaluates breathing and heart rate***

Prompt for observer: Show or say the baby is not breathing; heart is normal

| 1. Recognizes baby is not breathing but heart is normal | ☐ | ☐ |
| --- | --- | --- |
| 2. Continues Ventilation | ☐ | ☐ |

Prompt for observer: After 3 minutes show or say, “The heart rate is 120 per minute and the baby is breathing.”

| 1. Recognizes baby is breathing and heart rate is normal | ☐ | ☐ |
| --- | --- | --- |
| 1. Stops ventilation | ☐ | ☐ |
| 1. Monitors baby | ☐ | ☐ |
| 1. Communicates with mother | ☐ | ☐ |

**--------------------------------------------------------------------------------------------------------------------------**SCORING: Please add together the number of activities/ steps that were done and write it in the space below.

Number Done Correctly |____|____|

Data Collector’s code |____|____|

Were the following activities/ steps done? **Yes/ No**

**RECOGNIZES BABY IS NOT CRYING, VENTILATES AT 40 BREATHS/MINUTE, LOOKS FOR CHEST MOVEMENT, AND IMPROVES VENTILATION.**
