# Supplementary material for: Health facility service availability and readiness for intrapartum and immediate postpartum care in Malawi: A cross-sectional survey
Source: PLoS One. 2017 Mar 16;12(3):e0172492. doi: 10.1371/journal.pone.0172492 (PMC5354363; doi:10.1371/journal.pone.0172492)
Supplement: S1 Data — (ZIP) [file pone.0172492.s006.zip › Malawi HBB Eval_Tool 8 HBB Facility staffing and record_August 18 2012 FINAL.doc]

**MALAWI HBB EVALUATION**

**TOOL 8: FACILITY STAFFING and RECORD REVIEW**

**TO BE COMPLETED FOR EACH HEALTH FACILITY WITH MATERNITY/ LABOR AND DELIVERY UNIT**

**Name of Health Facility:**

**Facility Number: |_**C/I**_|__|__|__|	Date: |_**D**_|_**D**_ |_**M**_|_**M**_|_**Y**_|_**Y**_|**

**Observer Number |__|__|**

1. **Type of Health Facility (Tick (√ ) as appropriate):**

**i)	District Hospital **

**ii)	Health Center	**

iii) **CHAM Hospital	**
iv) **Private Hospital **
2. **Distance from this health facility to District Hospital/or Next Referral Hospital**

**Record in kilometers	km (One way)**

3. **Catchment Population of health facility:**
4. **Expected number of Women of Child Bearing Age (WCBA) of total population:**
5. **Staffing Available at Health Facility:**

| **Cadre of MNH service providers at Health Facility** | **Number allocated**  **to**  **Maternity** | **Number**  **trained in BEmONC or Newborn care** |
| --- | --- | --- |
| **Obstetrician/Gynaecologist** |  |  |
| **Doctor** |  |  |
| **Medical Assistant** |  |  |
| **Clinical Officer** |  |  |
| **Registered Midwife** |  |  |
| **Enrolled Nurse Midwife** |  |  |
| **Nurse Midwife Technician** |  |  |
| **Student** |  |  |
| **Other (specify cadre & record #s)** |  |  |

**PART B:**

**TO BE COMPLETED WITH THE REVIEW OF RECORDS FOR EACH FACILITY WITH MATERNITY/ LABOR and DELIVERY UNIT**

**Review the Labor & Delivery Record and obtain the following data**

**August 2011 to July 2012**

| **2011-2012**  **Month** | **Number**  **of**  **Total Deliveries** | **Number of Assisted Deliveries** | **Number**  **of**  **Caesarian Sections** | **Number**  **of**  **Maternal Deaths** |
| --- | --- | --- | --- | --- |
| **August 2011** |  |  |  |  |
| **September 2011** |  |  |  |  |
| **October 2011** |  |  |  |  |
| **November 2011** |  |  |  |  |
| **December 2011** |  |  |  |  |
| **January 2012** |  |  |  |  |
| **February 2012** |  |  |  |  |
| **March 2012** |  |  |  |  |
| **April 2012** |  |  |  |  |
| **May 2012** |  |  |  |  |
| **June 2012** |  |  |  |  |
| **July 2012** |  |  |  |  |
| **TOTAL** |  |  |  |  |

**Review the NEWBORN Record and obtain the following Data**

Instruction for data collector: If HBB register not available. Please extract data for column (D) from monthly register.

**August 2011 to July 2012**

| **2011-2012**  **Month** | **(A)**  **Number**  **of newborn deaths** | **(B)**  **Number**  **of**  **still births**  **Separate numbers & Add:**  **F** if Fresh SB  **M** if Macerated SB | | **(C)**  **Number of babies born with asphyxia** | **(D)**  **Number**  **of**  **babies with asphyxia resuscitated successfully**  **(ALIVE thereafter)** | **(E)**  **Number**  **of**  **babies with asphyxia referred to next level** |
| --- | --- | --- | --- | --- | --- | --- |
| **August 2011** |  | F |  |  |  |  |
|  |  | M  Tot |  |  |  |  |
| **September 2011** |  | F |  |  |  |  |
|  |  | M |  |  |  |  |
|  |  | Tot |  |  |  |  |
| **October 2011** |  | F |  |  |  |  |
|  |  | M |  |  |  |  |
|  |  | Tot |  |  |  |  |
| **November 2011** |  | F |  |  |  |  |
|  |  | M |  |  |  |  |
|  |  | Tot |  |  |  |  |
| **December 2011** |  | F |  |  |  |  |
|  |  | M |  |  |  |  |
|  |  | Tot |  |  |  |  |
| **January 2012** |  | F |  |  |  |  |
|  |  | M |  |  |  |  |
|  |  | Tot |  |  |  |  |
| **February 2012** |  | F |  |  |  |  |
|  |  | M |  |  |  |  |
|  |  | Tot |  |  |  |  |
| **March 2012** |  | F |  |  |  |  |
|  |  | M |  |  |  |  |
|  |  | Tot |  |  |  |  |
| **April 2012** |  | F |  |  |  |  |
|  |  | M |  |  |  |  |
|  |  | Tot |  |  |  |  |
| **May 2012** |  | F |  |  |  |  |
|  |  | M |  |  |  |  |
|  |  | Tot |  |  |  |  |
| **June 2012** |  | F |  |  |  |  |
|  |  | M |  |  |  |  |
|  |  | Tot |  |  |  |  |
| **July 2012** |  | F |  |  |  |  |
|  |  | M |  |  |  |  |
|  |  | Tot |  |  |  |  |
| **TOTAL** |  | F |  |  |  |  |
|  |  | M |  |  |  |  |
|  |  | Tot |  |  |  |  |

Indicate data source (s) used (Circle all that apply):

Maternity Register………………1

HBB Register………………..….2

Monthly facility report………….3

Other (specify): ___________ …8

If the HBB register is being used, please comment on the quality of the data (are all months and all data elements completed?)

______________________________________________________________________________

______________________________________________________________________________

______________________________________________________________________________

______________________________________________________________________________

______________________________________________________________________________
